# Development and Implementation of a Safety Incident Report System for Health Care Discipline Students During Clinical Internships: Observational Study

**DOI:** 10.2196/56879

**Published:** 2024-07-18

**Authors:** Eva Gil-Hernández, Irene Carrillo, Mercedes Guilabert, Elena Bohomol, Piedad C Serpa, Vanessa Ribeiro Neves, Maria Maluenda Martínez, Jimmy Martin-Delgado, Clara Pérez-Esteve, César Fernández, José Joaquín Mira

**Affiliations:** 1 Fundación para el Fomento de la Investigación Sanitaria y Biomédica (FISABIO) Alicante Spain; 2 Universidad Miguel Hernández Elche Spain; 3 Escola Paulista de Enfermagem, Universidade Federal de São Paulo São Paulo Brazil; 4 Clinical Management and Patient Safety Department Universidad de Santander Bucaramanga Colombia; 5 Biomedical Sciences Faculty Universidad Austral Pilar Argentina; 6 Instituto de Investigación e Innovación en Salud Integral, Facultad de Ciencias de la Salud, Universidad Católica de Santiago de Guayaquil Guayaquil Ecuador; 7 Hospital de Especialidades Alfredo Paulson, Junta de Beneficencia de Guayaquil Guayaquil Ecuador

**Keywords:** reporting systems, education, medical, nursing, undergraduate, patient safety

## Abstract

**Background:**

Patient safety is a fundamental aspect of health care practice across global health systems. Safe practices, which include incident reporting systems, have proven valuable in preventing the recurrence of safety incidents. However, the accessibility of this tool for health care discipline students is not consistent, limiting their acquisition of competencies. In addition, there is no tools to familiarize students with analyzing safety incidents. Gamification has emerged as an effective strategy in health care education.

**Objective:**

This study aims to develop an incident reporting system tailored to the specific needs of health care discipline students, named Safety Incident Report System for Students. Secondary objectives included studying the performance of different groups of students in the use of the platform and training them on the correct procedures for reporting.

**Methods:**

This was an observational study carried out in 3 phases. Phase 1 consisted of the development of the web-based platform and the incident registration form. For this purpose, systems already developed and in use in Spain were taken as a basis. During phase 2, a total of 223 students in medicine and nursing with clinical internships from universities in Argentina, Brazil, Colombia, Ecuador, and Spain received an introductory seminar and were given access to the platform. Phase 3 ran in parallel and involved evaluation and feedback of the reports received as well as the opportunity to submit the students’ opinion on the process. Descriptive statistics were obtained to gain information about the incidents, and mean comparisons by groups were performed to analyze the scores obtained.

**Results:**

The final form was divided into 9 sections and consisted of 48 questions that allowed for introducing data about the incident, its causes, and proposals for an improvement plan. The platform included a personal dashboard displaying submitted reports, average scores, progression, and score rankings. A total of 105 students participated, submitting 147 reports. Incidents were mainly reported in the hospital setting, with complications of care (87/346, 25.1%) and effects of medication or medical products (82/346, 23.7%) being predominant. The most repeated causes were related confusion, oversight, or distractions (49/147, 33.3%) and absence of process verification (44/147, 29.9%). Statistically significant differences were observed between the mean final scores received by country (*P*<.001) and sex (*P*=.006) but not by studies (*P*=.47). Overall, participants rated the experience of using the Safety Incident Report System for Students positively.

**Conclusions:**

This study presents an initial adaptation of reporting systems to suit the needs of students, introducing a guided and inspiring framework that has garnered positive acceptance among students. Through this endeavor, a pathway toward a safety culture within the faculty is established. A long-term follow-up would be desirable to check the real benefits of using the tool during education.

**Trial Registration:**

Trial Registration: ClinicalTrials.gov NCT05350345; https://clinicaltrials.gov/study/NCT05350345

## Introduction

### Background

Patient safety is an objective of health care practice in the health systems of all countries. However, the complexity and uncertainty that accompany health care makes this a practice not without risks. The World Health Organization leads the World Alliance for Patient Safety with the purpose of implementing safe practices and other actions with which to generate a safer environment in all health centers [1].

The information available regarding safety incidents focuses primarily on adverse events (AEs), which are incidents that result in harm to a patient. Slightly more than half of these AEs could have been prevented [2]. The results of research studies show that, in high-income countries, approximately 10% of patients admitted to hospitals experience an AE [3]. In primary and outpatient care, approximately 3% to 10% of patients experience an AE over the course of a year [4]. In 80% of cases, the damages are avoidable. In low- and middle-income countries, there are higher rates of AEs due to deficiencies in infrastructure, facilities, and accessibility [2]. So-called safe practices aim to reduce these figures and have proliferated across all countries [5]. Among them, incident reporting systems (IRSs) have emerged as a valuable tool to prevent safety incidents stemming from the same cause from recurring [2].

Studies indicate that up to 30% of students are involved in an AE during an academic year [6]. Moreover, during their internships, students observe decisions and procedures that may lead to errors or cause harm (AEs) to patients [7]. While access to IRSs is widespread in all health care systems, students of health care disciplines are often not adequately trained on how to use and benefit from these tools to create safer environments for patients. This lack of training restricts students’ acquisition of crucial competencies in several ways.

The familiarization of students with incident reporting addresses a significant educational practice gap. First, the absence of IRS exposure hinders students’ ability to understand what an incident report is, how to complete it, the extent of the information required, and how it functions to promote safer environments. This exposition to IRSs not only enhances their capability to effectively report incidents in future real-world contexts but also helps reduce the initial reluctance toward reporting. Second, reporting unsafe events can enhance practice and prevent future safety incidents. This active learning helps students identify and avoid recurring incidents by raising awareness of their causes. Third, providing students with access to IRSs raises awareness among future professionals of the critical importance of patient safety. It serves as a vital learning resource and offers an opportunity to change attitudes and foster the development of a proactive safety culture [8].

Despite this, the interventions designed and validated to achieve the goal of promoting incident reporting among health care discipline students are scarce [9]. There are also no tools to introduce these students to the analysis of the remote and immediate causes of safety incidents and the identification of barriers to prevent them from recurring. However, there are digital tools that are starting to be used to increase patient safety, particularly those based on gamification [10,11].

The effectiveness of gamification in health care education has been analyzed in several studies [12,13], showing improvements in knowledge, skills, satisfaction, behavior change, and attitudes compared to control groups. However, the usefulness of engaging health care discipline students in patient safety has not been assessed.

### Objectives

The primary objective of this study was to develop a patient safety IRS tailored to the needs of health care discipline students. The secondary objectives were to study the performance of different groups of students in the use of the platform and train them on the correct procedures for reporting.

## Methods

### Study Design

**Figure 1:**
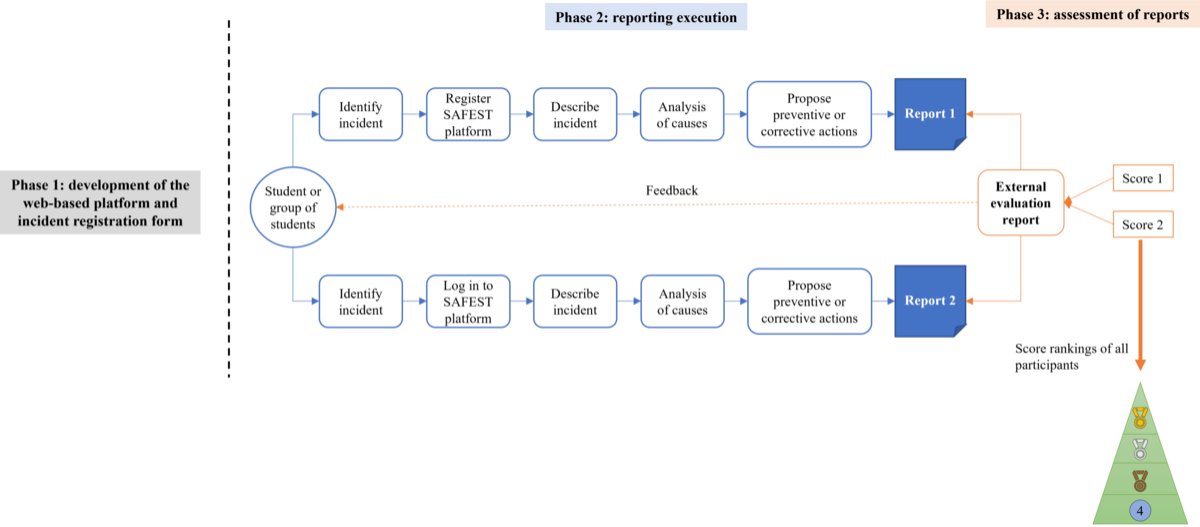
Workflow of the study divided into the 3 phases. SAFEST: Safety Incident Report System for Students.

This was an observational study developed in 3 phases (Figure 1), in which safety incident reports made by final-year students in medicine and nursing during their clinical internships were analyzed. The students were enrolled in universities from Argentina, Brazil, Colombia, Ecuador, and Spain once they had gained experience from their clinical placements. All these universities are members of the European Researchers’ Network Working on Second Victims Consortium, with the Latin American ones as third-party or observer countries and Spain as the promoter of the network.

In the participating countries, medical studies are typically completed over 6 years, with the last 3 years progressively incorporating more clinical practice. However, nursing studies exhibit greater variability and can range from 4 to 6 years in duration. In these programs, the final year is usually dedicated to clinical practice. Nursing curricula also show more diversity in their content, with some programs focusing more on hospital-based activities whereas others emphasize community health practice. Nonetheless, due to international guidelines on required competencies and clinical practice hours, these programs are standardized to ensure consistency in training.

In countries such as Argentina, Brazil, Chile, Ecuador, and Spain, medical and nursing programs follow this general structure but with some national variations. For example, in Spain, medical students undergo a rigorous 6-year program with a strong emphasis on clinical rotations in the later years. Nursing programs in Spain typically last 4 years, with the final year focused on intensive clinical practice. In Brazil and Argentina, similar patterns are observed, although the specifics of the curriculum and clinical exposure may differ slightly due to local health care needs and educational frameworks. As in other places, teaching patient safety is limited, representing one of the gaps highlighted in various studies [14].

This study is reported according to the STROBE (Strengthening the Reporting of Observational Studies in Epidemiology) guidelines for cross-sectional studies ([15]; see Multimedia Appendix 1).

### Phase 1: Development of the Web-Based Platform and Incident Registration Form

This phase consisted of the development of the web-based platform and the incident registration form, named Safety Incident Report System for Students (SAFEST).

To design the content for the incident registration form, various existing systems at different levels were used as references. The existing systems in health care centers require users to be part of the center’s staff, making them inaccessible to other groups, including students. In addition, while these systems collect the reporter’s assessment of potential causes, they do not advance to propose alternatives for preventing future safety incidents. This aspect of our educational initiative is crucial to influencing students’ attitudes toward safety reporting. Due to these limitations, the available safety systems in the health care facilities were not suitable for student practice and, therefore, deemed inappropriate for our purposes. Consequently, we decided to design a new system that closely mimics the systems that students will encounter in their professional practice. This approach ensures that students receive relevant and practical training, enhancing their ability to effectively report and analyze safety incidents in the future.

A database was then constructed incorporating fields gathered from the Patient Safety Reporting and Learning System of the Spanish National Health System [16]; the Adverse Event Reporting and Registration System of the Valencia Health Agency [17]; and Based on Root Cause Analysis (BACRA) [18,19], a web-based application based on root cause analysis and failure mode and effects analysis.

One of the key features of SAFEST is that each section and registration field (eg, center type, care complications, damage type, or care received after the incident) offered an extensive range of response options in different formats (single-select drop-down menu or multiple-choice answer). This design facilitated the reporting task for the students as they rarely needed to use natural language to describe a situation. This approach aligns with the latest advancements in reporting systems, minimizing errors in subsequent coding while providing a comprehensive catalog of options. However, in some cases in which the preset options may limit the recording, students can add a qualitative description to complement the recording. For example, when describing the incident, the student should characterize the event according to the classic typification of its nature, that is, whether the origin of the incident was related to complications of care, care-related infection, effects of medication or medical devices, complications of a procedure, or other situations not covered by the previous categories (eg, unexpected death of the patient). All these categories are detailed in a list of possibilities in a multiple-choice format. However, in all categories (including “Other”), the student may choose a final option as “Other,” in which case they should describe in words the situation in question. The first version of the database was created in Spanish.

From this database, common and specific aspects of each form were identified, and a preliminary draft of the proposal was developed accordingly. This draft underwent review by 3 subject matter experts from different Latin American countries, and the resulting changes and suggestions were incorporated to produce a high-quality form. This latest version of the tool was translated into English by EB and VRN, both of whom use the 2 languages regularly in the academic setting, ensuring the equivalence of the versions through back translation. The necessary modifications to ensure the adequacy of the system were made. Simultaneously, the visual identity and acronym for the platform were developed (Multimedia Appendix 2).

### Phase 2: Introduction Seminar and Incident Reporting Execution

#### Overview

During this phase, students received an introductory seminar on patient safety and reporting and were given access to the platform. These introductory seminars were conducted by the responsible coordinators from the 5 universities (1 from each country; see Multimedia Appendix 3 for the educational materials used during the seminars). During these seminars, the project and the platform were presented, and attendees were given the opportunity to ask any questions they had.

The seminars contributed to the recruitment of participants based on voluntary participation without offering any academic grade advantages. To incentivize student engagement, they were provided with the opportunity to obtain a Miguel Hernández University nanocourse certificate. Moreover, the highest scores qualified for a draw with 4 new smartwatches as the prize, thus incorporating classic elements of gamification strategies. Of the smartwatches, 2 were assigned to the people with the highest and second-best scores and who had also submitted their feedback, whereas the other 2 were drawn among all reports with a score of >3.0 and who had also completed their feedback.

The specific instruction given to the students was to report any safety incidents that had occurred in their training health care center and of which they were aware, either because they had been involved or because they were witnesses. To introduce students to this exercise and standardize explanations and instructions on how to respond, concise use instructions were created along with video tutorials on navigating the website and submitting reports and a schematic diagram of the operation (Multimedia Appendix 4). The same presentation was used in all countries. In accordance with the academic calendars of the participating countries, the report submission period spanned from September 14, 2022, the day when the first seminar was held, to November 8, 2023.

#### Participants

Medicine (n=176) and nursing (n=47) students who had completed more than half of their educational program and were performing clinical internships were invited to participate. Recruitment was conducted by the professor in charge in each country with students in the corresponding academic years who met the selection criteria.

#### Study Size

According to existing literature, in pilot studies, if a problem exists with a 5% probability in a potential study participant, a sample size of 59 participants will almost certainly identify the problem with 95% confidence [20].

### Phase 3: Assessment of Reports and Feedback on the Experience

#### Feedback

This phase ran in parallel to the previous one and involved external evaluation and feedback on the reports received that could prove useful for the students improvement in continuing to send reports. In total, 2 independent assessments were conducted for each incident report by members of the platform’s promoting team. As a final exercise, students who had submitted at least 1 report were invited 1 month after this activity was over to fill out a satisfaction questionnaire.

#### Data Sources

The information provided in this study stems from the firsthand experiences of each student.

#### Variables

The outcomes we aimed to assess were the students’ performance in reporting using the platform, which includes an estimation of potential causes to raise awareness of the inherent risks in health care activities, and their satisfaction with the experience.

To study their ability in reporting, a rubric (Table 1) was followed, in which the 2 reviewers independently rated the information provided about the incident, the analysis of immediate and latent causes, and the corrective or preventive plan proposed by the student using a scale of 1 to 5 points for each one, where the higher the score, the better the assessment. In addition, strengths and areas for improvement were included in the evaluation as an open-text field. The individual score from each evaluator was obtained by calculating the arithmetic mean of these 3 points. The final score for that report was the average of the 2 scores obtained from each evaluator.

**Table 1:** Rubric designed to assess the correctness of the reports made by students.

Points		Description
**To what extent is the information complete and descriptive enough?**		
	2 points	The provided information allows for the understanding of the events.
	2 points	The information is consistent throughout the entire report.
	1 point	All fields are properly filled out.
**To what extent is the analysis of immediate and latent causes complete and adequate?**		
	2 points	The provided information is comprehensive, and reasons with a high probability of influence are not overlooked.
	2 points	The provided information offers sufficient details to envision the scenario of what happened.
	1 point	The selected information is logical and does not appear to have been chosen merely for completion.
**To what extent the corrective or preventive plan proposed is realistic and responds to the problem?**		
	1 point	The plan has a corrective or preventive nature.
	1 point	The proposed plan is realistic.
	1 point	The proposed plan is understandable.
	1 point	Details are addressed to implement the proposed plan.
	1 point	Language and spelling are appropriate.

To analyze their satisfaction with the experience, they were asked to complete a questionnaire with 3 aspects to rate on a scale of 1 to 5, with 1 being *not at all* and 5 being *very much*: “Do you believe that after this experience you would be capable of generating reports accurately?” (question 1), “Has viewing the assessments and comments you received on your reports been beneficial for your learning?” (question 2), and “Have you felt confident in terms of the privacy and anonymity of your reports?” (question 3). In addition, they had a text field available to input any suggestions that could contribute to improving the platform (question 4).

The independent variables used included the country from which the report was made, the sex of the reporter, their ongoing studies, and the number of internship hours completed up to the moment of reporting. All these data were incorporated into the incident registration form itself.

#### Bias

When the form was sent to the partners for review, a language check was also requested to address any idiomatic barriers that may have existed to allow for cross-cultural conclusions of the study and avoid possible biases related to linguistic nuances. Cultural differences were also considered, ensuring that items were comparable across countries.

#### Statistical Methods

To gain a comprehensive understanding of the reported incidents, descriptive analyses were conducted. To obtain the results of the phase of assessment, various statistical analyses were conducted. Descriptive statistics were computed for each of the 3 dimensions under analysis as well as for the overall score, with stratification by country, sex, and educational background. The weighted Cohen κ was computed to evaluate the agreement among the scores assigned by different pairs of evaluators for each dimension. Before proceeding with the analysis of the final scores of each report, the normality of the sample was assessed using *Q*-*Q* plots and the Shapiro-Wilk normality test. The relationship between the number of internship hours and the final score was examined using the Spearman correlation coefficient. Finally, differences in scores among countries, sexes, and educational backgrounds were investigated using the nonparametric Kruskal-Wallis test and the Mann-Whitney *U* test. The *P* value significance was set at .05. Data analyses were performed using SPSS Statistics (version 28.0.0; IBM Corp).

### Ethical Considerations

This study was authorized by the Research Ethics Committee of Sant Joan d’Alacant University Hospital (22/027) and registered on ClinicalTrials.gov (NCT05350345).

Informed consent for study participation was obtained at the time of registration on the platform, whereby individuals were required to select the corresponding checkbox with instructions provided regarding the process for revoking their participation. After reporting, the report was automatically encoded with a numerical identifier by the platform. Throughout the assessment process, participant sociodemographic data were concealed to ensure evaluator objectivity.

No form of financial compensation was provided for participation or recruitment.

## Results

### Phase 1: Development of the Web-Based Platform and Incident Registration Form

SAFEST [21] and the servers were located in Miguel Hernández of Elche University (Spain). Participation was allowed both individually and in groups of 2 to 3 students.

**Figure 2:**
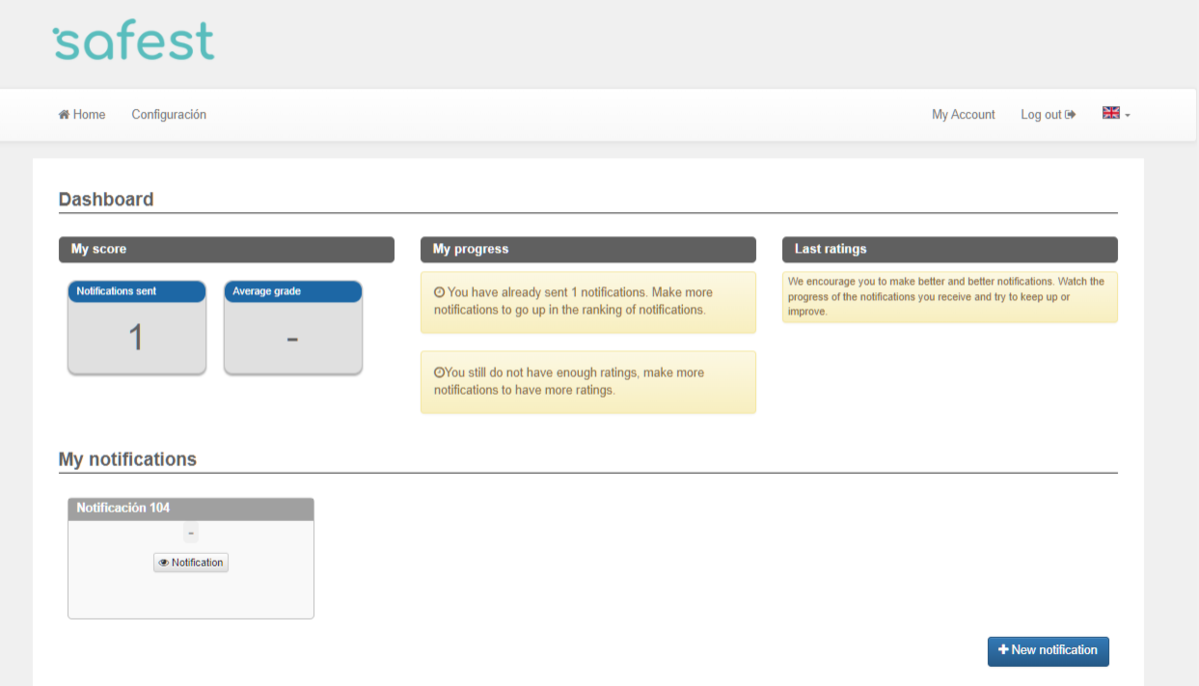
Appearance of the Safety Incident Report System for Students (SAFEST) platform dashboard.

When accessing the page, users could find an explanatory text about the project, logos of collaborators, and buttons to access the platform or register. Upon initial access, the user was required to provide consent to participate in the study. The platform was available in both Spanish and English. Once logged in, the dashboard was shown (Figure 2), where they could view the total of submitted reports, their average score, and the progression of their results, as well as their position in the score ranking at any time. In addition, they had access to previously submitted reports, as well as the button to access the incident registration form.

The final form was divided into 9 sections: data of the reporting center, patient data, notifier data, incident data, description of the incident, damage assessment, factors influencing the incident, care received after the incident, and reflections.

In total, it consisted of 48 questions (distributed as depicted in Textbox 1) that allowed for obtaining the necessary information about the incident, conducting an analysis of the causes, and proposing corrective or preventive actions. The complete form can be found in Multimedia Appendix 5.

**Questions asked on the reporting form and types of responses.**

**Data of the reporting center**

- Center type (drop-down menu)

**Patient data**

- Patient’s age (drop-down menu)
- Patient’s sex (drop-down menu)
- Patient’s risk factors (multiple choice)

**Notifier data**

- Notifier’s sex (closed-ended question)
- Country from which the notification was made (drop-down menu)
- Studies in the course (drop-down menu)
- Institution (drop-down menu)
- Year (closed-ended question)
- Internship hours carried out so far in that department (open-ended question)

**Incident data**

- Date of the incident (date)
- Time of the incident (time)
- Date of the notification (date)
- Time of the notification (time)
- Where it took place (drop-down menu)
- Number of people related to the incident (open-ended question)
- Position or positions of the person or people involved (multiple choice)
- Frequency or probability of recurrence (drop-down menu)
- Participation in the incident (drop-down menu)

**Description of the incident**

- Care complications (multiple choice)
- Care-related infection (multiple choice)
- Effects of medication or medical products (multiple choice)
- Complications of a procedure (multiple choice)
- Other (multiple choice)

**Damage assessment**

- Damage type (drop-down menu)
- Severity (drop-down menu)
- Patient autonomy (drop-down menu)
- Estimation of the damage duration (drop-down menu)

**Factors that conditioned the incident**

- Patient or family factors (multiple choice)
- Equipment and resource factors (multiple choice)
- Individual factors of the health care professional or professionals (multiple choice)
- Work environment factors (multiple choice)
- Oral and written communication between professionals factors (multiple choice)
- Patient communication factors (multiple choice)
- Teamwork and leadership factors (multiple choice)
- Task-related factors (multiple choice)
- Organizational and management factors (multiple choice)
- Other factors (open-ended question)

**Care received after the incident**

- Care received after the incident (multiple choice)

**Reflections**

- Has the center been notified? (drop-down menu)
- Could the incident have been prevented? (drop-down menu)
- How could it have been prevented? (open-ended question)
- How could the probability of occurrence or the severity of this event be reduced? (open-ended question)
- To what extent was all the information necessary to analyze the causes of the event available? (open-ended question)
- Have measures been put in place to prevent it from happening in the future? (drop-down menu)
- What measures have been put in place to prevent it from happening in the future? (open-ended question)
- Do you consider that the analysis could have been different if you had had access to another source of information? (open-ended question)
- Write here any other comments you may have (open-ended question)

To streamline the use of the system, selection questions and drop-down menus were used to report incidents. Both the *Description of the incident* and *Factors that conditioned the incident* blocks allowed for more than one option to be selected. Written input was only necessary in the *Reflections* part.

### Phase 2: Introduction Seminar and Incident Reporting Execution

A total of 105 students from the 5 countries participated voluntarily and actively by submitting at least 1 report (participation rate: 105/223, 47.1%). By country, this corresponds to 16.2% (17/105) of students from Argentina, 12.4% (13/105) of students from Brazil, 10.5% (11/105) of students from Colombia, 32.4% (34/105) of students from Ecuador, and 28.6% (30/105) of students from Spain. Of the 105 participants, 35 (33.3%) were male, 68 (64.8%) were female, and 2 (1.9%) specified their sex as *other*. Only 1.9% (2/105) of them formed a team. Regarding their studies, 66.7% (70/105) were pursuing a degree in medicine, 28.6% (30/105) were enrolled in nursing studies, 1.9% (2/105) were part of the pediatric specialization program, 1.9% (2/105) were students from the radiology and diagnostic imaging specialization program, and 1% (1/105) belonged to the orthopedics and traumatology specialization program.

A total of 147 reports were submitted as 14 users provided >1 report. Of the 147 received reports, 18 (12.2%) were from Argentina, 13 (8.8%) were from Brazil, 44 (29.9%) were from Colombia, 35 (23.8%) were from Ecuador, and 37 (25.2%) were from Spain.

Of the 147 safety incident reports, a substantial majority, specifically, 144 (98%) reports, occurred in a health care setting, with most occurring in a hospital context (n=132, 89.8%). Within this hospital-centric subset, most incidents were concentrated in hospitalization units (45/132, 34.1%). Other noteworthy locations included surgical block areas (21/132, 15.9%), emergency departments (17/132, 12.9%), support services (14/132, 10.6%), day hospitals (12/132, 9.1%), and intensive care units (10/132, 7.6%).

Regarding the nature of the incidents, Table 2 illustrates the frequency with which each major classification category was selected. On most occasions, events from different blocks were registered in the same report.

**Table 2:** Nature of the reported safety incidents (n=346).

Type of incident	Reports, n (%)
Care complications	87 (25.1)
Effects of medication or medical products	82 (23.7)
Complications of a procedure	67 (19.4)
Other	60 (17.3)
Care-related infection	50 (14.5)

Specifically, from the available list of the most common safety events included in SAFEST (drop-down list), students’ reports were related to “Worse evolutionary course of the main pathology” (27/147, 18.4%), “No harm” (24/147, 16.3%), “Ineffective analgesia-related pain” (19/147, 12.9%), “Falls and consequent fractures” (18/147, 12.2%), “Surgical site or traumatic wound infection” (15/147, 10.2%), “Contusion” (14/147, 9.5%), “Unexpected death” (14/147, 9.5%), “Headache” (13/147, 8.8%), and “Prescription error” (13/147, 8.8%).

The reported causes are shown in Table 3. According to the number of reports in which they appear, we established the following categories: “Patient or family factors” (112/147, 76.2% of reports), “Equipment and resource factors” (71/147, 48.3% of reports), “Individual factors of the healthcare professional(s)” (118/147, 80.3% of reports), “Work environment factors” (103/147, 70.1% of reports), “Oral and written communication between professionals factors” (76/147, 51.7% of reports), “Patient communication factors” (64/147, 43.5% of reports), “Teamwork and leadership factors” (93/147, 63.3% of reports), “Task-related factors” (91/147, 61.9% of reports), and “Organizational and management factors” (81/147, 55.1% of reports).

**Table 3:** Causes and contributing factors of the incidents grouped by category (n=147).

Factor			Reports, n (%)
**Patient or family factors**			
	Comorbidity or complexity of the condition	33 (22.4)	
	Low economic level	30 (20.4)	
	Noncooperative attitude (noncompliance)	35 (23.8)	
	Lack of family or support networks	40 (27.2)	
	Poor communication with relatives	18 (12.2)	
	Altered cognitive status	8 (5.4)	
	Does not provide correct or enough information	14 (9.5)	
	Recent surgery	15 (10.2)	
	Educational and social factors to consider	20 (13.6)	
	Other patient factors	18 (12.2)	
	Mental disorder	3 (2)	
**Equipment and resource factors**			
	Improper storage or accessibility	19 (12.9)	
	Malfunctions	14 (9.5)	
	Equipment maintenance issues	12 (8.2)	
	Lack of alternative materials	11 (7.5)	
	Incorrect labeling	11 (7.5)	
	Equipment deficit (including nonsterile material)	10 (6.8)	
	Inadequate resource design (eg, bell)	10 (6.8)	
	Product or drug unavailability	10 (6.8)	
	Improper calibration	9 (6.1)	
	Nonstandard equipment	7 (4.8)	
	New equipment or resource	5 (3.4)	
	Failure to access or unavailability of the digital medical record	4 (2.7)	
	Expiration	2 (1.4)	
	Similar container or name	2 (1.4)	
**Individual factors of the health care professional or professionals**			
	Confusion, oversight, or distractions	49 (33.3)	
	Overload or work pressure	37 (25.2)	
	Lack of knowledge of regulations or protocols of performance	25 (17)	
	Uncooperative attitude	24 (16.3)	
	Inadequate or insufficient anamnesis, examination, or tests	23 (15.6)	
	Medication error (prescription or dispensing)	21 (14.3)	
	Inadequate or insufficient knowledge or skills	20 (13.6)	
	Low motivation	15 (10.2)	
	Diagnostic error	13 (8.8)	
	Inadequate or insufficient training	13 (8.8)	
	Not verifying the treatment that the patient is currently taking	12 (8.2)	
	Little experience in the workplace	10 (6.8)	
	Inadequate timetable	8 (5.4)	
	Inappropriate interpretation of analytical or test results	5 (3.4)	
**Work environment factors**			
	Distractions in the environment	38 (25.9)	
	Shift-related fatigue	36 (24.5)	
	High care pressure	29 (19.7)	
	Inadequate environment—cleaning, beds, or space	26 (17.7)	
	Inadequate environment—noise, light, or temperature	19 (12.9)	
	Performance of outside tasks	14 (9.5)	
	Excessive staff turnover or inexperience	12 (8.2)	
	Inadequate staff-to-patient ratio	11 (7.5)	
	Security and access to restricted areas	2 (1.4)	
**Oral and written communication between professionals factors**			
	The information does not reach the entire team	30 (20.4)	
	Ambiguous verbal indications	23 (15.6)	
	Insufficient or inadequate records	20 (13.6)	
	Using an inappropriate channel	19 (12.9)	
	Inappropriate body language	15 (10.2)	
	Incorrect use of language	11 (7.5)	
**Patient communication factors**			
	Insufficient or inadequate records	23 (15.6)	
	Ambiguous verbal indications	17 (11.6)	
	Using an inappropriate channel	16 (10.9)	
	Incorrect use of language	14 (9.5)	
	Inappropriate body language	12 (8.2)	
	Language barrier	6 (4.1)	
**Teamwork and leadership factors**			
	Lack of coordination in the team	40 (27.2)	
	Inadequate supervision	40 (27.2)	
	Low risk awareness	31 (21.1)	
	Inaccurate assignment of tasks	20 (13.6)	
	Conflict between team members	12 (8.2)	
	No effective leadership	9 (6.1)	
**Task-related factors**			
	Absence of process verification	44 (29.9)	
	Unknown protocol or noncompliance	30 (20.4)	
	Absence of guidelines or protocols	19 (12.9)	
	Inadequate or outdated protocol	18 (12.2)	
	Too complex task	7 (4.8)	
**Organizational and management factors**			
	Absence of evaluation systems	16 (10.9)	
	Error in health information	16 (10.9)	
	Nonexistent or inadequate risk management	16 (10.9)	
	Error in medical documentation	12 (8.2)	
	Insufficient organizational structure	12 (8.2)	
	Absence of support mechanisms in a risk situation	11 (7.5)	
	Incorrect patient identification	11 (7.5)	
	Insufficient deployment of a proactive security culture	11 (7.5)	
	Delays in the performance of tests or interconsultations	9 (6.1)	
	Insufficient care structure	9 (6.1)	
	Wrong appointment or scheduling	7 (4.8)	
	Gaps or failures in the information system	6 (4.1)	
	Inadequate or nonexistent treatment plan	5 (3.4)	
	Long waiting list	5 (3.4)	

When asked about whether the event had been reported at the center, in 41.5% (61/147) of the reports the answer was “Yes”; in 34.7% (51/147) of the reports, the answer was “I don’t know”; and, in 23.8% (35/147) of the reports, the answer was “No.” Finally, 93.9% (138/147) of the reported events were classified as preventable compared to 6.1% (9/147) that were categorized as nonpreventable.

### Phase 3: Assessment of Reports and Feedback on the Experience

Considering the 147 reports received, the mean final score obtained was 3.40 (SD 0.92) out of 5, and 111 (75.5%) reports had a final score of ≥3.0. For each of the 3 aspects studied, an average score of 3.38 (SD 1.29) was obtained for the section on giving information about the incident, an average score of 3.54 (SD 1.21) was obtained for the analysis of causes, and an average score of 3.30 (SD 1.30) was obtained for the proposal of a corrective or preventive plan. Table 4 shows the means of the final scores segregated by category.

**Table 4:** Mean final scores segregated by country, sex, and studies.

Variables			Values, mean (SD)
**Country**			
	Argentina	3.66 (0.89)	
	Brazil	3.65 (0.75)	
	Colombia	3.28 (0.88)	
	Ecuador	2.81 (0.88)	
	Spain	3.89 (0.73)	
**Sex**			
	Male	3.06 (0.99)	
	Female	3.64 (0.84)	
	Team	3.46 (0.60)	
	Other	3.58 (0.42)	
**Studies**			
	Medicine	3.34 (0.95)	
	Nursing	3.66 (0.82)	
	Pediatric specialization	3.25 (0.92)	
	Radiology and diagnostic imaging specialization	3.55 (0.59)	
	Orthopedics and traumatology specialization	2.75 (1.30)	

Significant differences were found in the final scores based on country (*P*<.001) and sex (*P*=.006). However, no significant results were obtained when comparing scores based on studies (*P*=.47). Similarly, when focusing on the 2 main groups (medicine and nursing), there were no significant differences (*P*=.11). Comparisons by groups for the significant variables are presented in Tables 5 and 6.

**Table 5:** *P* values for final score mean comparisons (country).

Country	Argentina	Brazil	Colombia	Ecuador	Spain
Argentina	—^a^	.92	.15	.004	.40
Brazil	.92	—	.19	.004	.36
Colombia	.15	.19	—	.01	.003
Ecuador	.004	.004	.01	—	<.001
Spain	.40	.36	.003	<.001	—

^a^Not applicable.

**Table 6:** *P* values for final score mean comparisons (sex).

Sex	Male	Female	Other	Team
Male	—^a^	.001	.44	.25
Female	.001	—	.82	.33
Other	.44	.82	—	.69
Team	.25	.33	.69	—

^a^Not applicable.

Regarding the internship hours carried out in that department, no correlation was found with the score obtained on each report (–0.079; *P*=.18). Finally, the interrater agreement analyses revealed consistency between each pair of evaluators across all cases (Table 7).

**Table 7:** Weighted Cohen κ values obtained for each pair of evaluators.

	Pair 1		Pair 2	
	Cohen κ	*P* value	Cohen κ	*P* value

Complete and descriptive information	0.324	<.001	0.304	<.001
Analysis of immediate and latent causes	0.420	<.001	0.195	.009
Corrective or preventive plan	0.344	<.001	0.258	<.001

A total of 15 students participated in discussing the experience through the satisfaction questionnaire, providing an average rating of 4.06 (SD 1.00) for question 1, an average rating of 4.18 (SD 1.22) for question 2, and an average rating of 4.56 (SD 1.09) for question 3. Moreover, they provided the following feedback in the improvement suggestion section: “The platform has been very useful to me, and I suggest that similar projects continue to be conducted virtually to encourage widespread participation,” “It would be beneficial if the platform allows the upload of images as evidence for each incident,” and “Consider incorporating a text comment box, allowing individuals involved in the incident to narrate the events rather than solely selecting an option. This would prevent overlooking crucial details that might be of interest for subsequent management and error prevention.”

## Discussion

### Principal Findings

A platform named SAFEST was developed, allowing students to submit reports and receive feedback regarding provided information, causal analysis, and proposed improvements. The educational practice simulates the environment they will encounter in their professional practice regarding the reporting of safety incidents. Students from 5 countries participated in this initiative, sending reports in which most incidents occurred within hospital settings and involved complications related to care and medication.

The identified causes of safety events reported by students using SAFEST included confusion, oversight, or distractions and absence of process verification. Across all countries, the average score exceeded 2.5, although significant differences in average scores among some countries are observed. Overall, the experience was highly regarded.

This paper delves into a comprehensive portrayal of the SAFEST platform, focusing on its inception, development, and implementation. The SAFEST platform stands as a pivotal reporting system strategically crafted to initiate health care discipline students into the realm of identifying and reporting current safety lapses within health care environments. In addition, this paper describes the perception that medical and nursing students had regarding incidents impacting patient safety, their attributed causes, and potential preventive or corrective measures. This reflection on what they identified as incidents can provide professors with feedback for planning their teaching.

The platform and designed materials allowed medical and nursing students to be introduced to safety incident reporting. SAFEST recreated the natural context in which reporting occurs, providing students with an experience close to reality but facilitating the process by allowing for step-by-step guided reporting. The IRS form follows the same structure and covers the same fields as those available for health care professionals as it was developed based on 3 existing systems. However, there are differences in terms of approach or responsibility. Students report situations that they observe or are involved in but are not the authors of. In addition, they encounter disparities regarding the accessibility of information for cause analysis as they do not have full access to the patient’s medical history. The gathered information is not disseminated, nor does it bring consequences for the involved parties. This type of active learning can also facilitate a better understanding of the impact of safety incidents and their causes. By doing this, students can grasp the basic concepts of fostering a proactive safety culture for patients. Once in clinical settings as professionals, they will be able to overcome the natural barrier hindering reporting through their participation in SAFEST. They will also have gained experience in analyzing both the immediate and remote causes of safety incidents and identifying preventive or corrective measures. These types of educational interventions prompt reflection on how errors occur in clinical practice, aiding in distinguishing between honest mistakes and intentional errors. If specific patient safety content is taught during regular classes while conducting the reporting practice, one can expect a greater impact of this practice on instrumental and attitudinal competencies. This aspect should be verified in the future.

Notably, the existing literature predominantly reflects studies conducted within the field of nursing [7,8,22], leaving a noteworthy void in the examination of reporting mechanisms across broader health sciences education. Breaking away from this convention, our research introduces a groundbreaking element not only by incorporating medical students into its purview but also by providing a system that can be extended to other disciplines that develop their practices in the clinical field.

Moreover, the provision of feedback helps students learn and improve their skills. Similarly, the integration of a gamified environment adds an element of engagement and motivation to the learning process. By incorporating elements of game design such as rewards and progression systems, the learning experience is transformed into a user-friendly practice.

Reporting systems constitute one of the fundamental tools for creating increasingly safe environments for patients [2]. It has been demonstrated that they also have a positive impact on the safety culture within health care institutions. However, students in health care disciplines typically become familiar with this tool once they are in health care settings either as residents or professionals. Simulation, as portrayed in this case, stands as one of the most used approaches in teaching-learning methods [23]. The approach of this exercise ensures active student engagement in reporting. The feedback provided to the students facilitated the enhancement of their proficiency and enabled them to report accurately. This aspect has been highlighted as significant in other studies [24].

The data from this study suggest that introducing a practice on how to report and why it is important was well received by the participants in this academic exercise, resulting in reports of suitable quality. Previous studies [25] have suggested that students demonstrate enhanced proficiency in detecting and analyzing incidents when they are not involved in them. Therefore, incorporating a reporting exercise during their internship period would contribute to cultivating a patient safety culture among students. This approach facilitates experiential learning, enabling students to comprehend the intricacies of incidents, empowering them to identify and mitigate such occurrences in their future professional endeavors.

The incident reporting by students has 2 strengths: the firsthand experience in clinical risk management within a health care institution and the provision of specific information that can contribute to enhancing comprehensive patient safety education among students. When delving into the results obtained in terms of scores, we found congruence with the results of other studies [25] in that the analysis of causes emerged as the strongest aspect, whereas the proposal of an improvement plan proved to be the weakest. This is particularly evident in cases in which patient safety content was integrated into the curriculum, where greater familiarity with patient safety was correlated with higher-quality reporting. The variations in the scores obtained can be explained by the curricular differences between each country. The Argentinean university involved offers 2 subjects on patient safety during the 5 years of the degree. It also has a patient safety program in which theoretical, simulation, and practical modules on patient safety (international goals and risk management) are offered so that students receive training throughout their degree, from first to fifth year, in all subjects that involve field practice. In contrast, the Ecuadorian university involved lacks any specific courses on the subject during the 6 years. Spanish students receive specific lectures on patient safety in 4 subjects starting in the second year before entering the internship in the sixth year. One of these subjects also incorporates specific topics on AEs and their communication. In the Colombian university involved, there is no specific subject in the curriculum dedicated to this matter in the first 5 years of study. However, before engaging in clinical internships in the final year, students are required to complete a course on clinical management and health, which delves into introductory topics related to patient safety. In the case of the Brazilian university, the term “patient safety” is explicitly referenced in the curriculum of 6 subjects, spread out from the second to the fourth year of studies. Moreover, in another 17 subjects, while there may not be an explicit mention, faculty members address the subject matter throughout the duration of the academic term.

Similarly, female students achieved higher scores in the evaluation of their reports. Nevertheless, there is no existing literature to substantiate this observation, prompting the need to consider the influence of other factors that could account for it. In our case, these outcomes might be influenced by the sample distribution as reports submitted by male students were predominantly concentrated in Colombia (23/147, 15.6%) and Ecuador (21/147, 14.3%), the 2 countries exhibiting the lowest mean scores. Therefore, these differences might not be explained solely by sex but rather by the background in patient safety.

In this study, 47.1% (105/223) of students participated submitting at least one report. This percentage contrasted with the findings of other studies, which reported participation rates of approximately 12% [26]. This increase opens the door to a more in-depth exploration of the factors that may be influencing this elevated level of student participation. One potential line of inquiry focuses on student motivation and how it may be linked to the design or implementation of the reporting system. Examining the effectiveness of strategies used to encourage participation could shed light on the dynamics that lead to more active engagement by students in this particular context.

It is not surprising that many incidents took place in hospitals. First, students from both disciplines undertook most of their practical training in this environment, and this clinical exposure increases the likelihood of witnessing or being involved in safety incidents. Moreover, hospitals typically handle more complex and critical cases compared to other health care settings as well as conducting a greater quantity and variety of procedures. However, this figure may be influenced by the students’ risk perception. It is plausible that primary care settings are perceived as less prone to safety incidents, leading students to pay less attention to their surroundings in such environments. In analogous studies, the most frequently reported type of incident was associated with medication administration [7]. However, in our case, what emerged most frequently throughout the reports were incidents related to caregiving. This outcome is likely related to the information more readily accessible to students, explaining why they witness fewer medication errors than expected during their practice [22].

During their clinical placements in health care settings, students frequently witness safety incidents of different severities, triggering conflicting emotions—from fear of speaking up to guilt for remaining silent. Studies suggest that approximately 4 out of every 10 students in training admit to having made at least one medical error during their training period [27]. Most of these errors involve lapses in clinical judgment (7 out of 10 cases). The primary causes of these errors have been associated with deficiencies in supervision and in the students’ own technical competencies [28]. They often feel that the causes of these events are not adequately addressed. Once the practice session ends, they are left without information on whether the incident was reported, whether its causes were analyzed, or whether any subsequent actions were taken, all of which they might be unaware of. The attitudes and coping strategies of nursing students following the recognition of a medical error have been explored [29-31]. On the basis of our understanding, students who become implicated in an AE or near-miss situation tend to manifest symptoms aligned with the experience of second victims [27,32]. Familiarizing themselves with reporting and analyzing incident causes offers them a new perspective that we can also expect to aid them emotionally.

Finally, following the suggestions provided by the students and, thus, incorporating user-centered design principles, we found it highly beneficial to incorporate a text box for a brief narrative of the events. We believe that the optimal approach would involve presenting a comprehensive set of options to encourage reflection, prompting individuals to consider aspects they might not have otherwise. Subsequently, a field will be provided for participants to describe the unfolding of events in their own words. This aligns with the findings of King et al [33], who advocate for a balanced approach in future patient reporting systems, integrating closed-ended questions for cause analysis and classification alongside open-ended narratives to accommodate patients’ potential limitations in understanding terminology.

### Implications of Findings

By providing a guided process, students are aided in considering a variety of factors that could pose potential risks, ranging from material resource deficiencies to patient attitudes or workload overload. Moreover, they learn to analyze different variables, weigh consequences, and make informed decisions based on available information. Consequently, students acquire skills and experience that they are expected to be able to apply in similar situations in the future. Similarly, by increasing awareness of risks and sources of mistakes and empowering students to identify them, the likelihood of involvement in dangerous or problematic situations is expected to be reduced [34], thereby contributing to the creation of safer environments.

The apprehension surrounding potential negative outcomes of reporting has been present since the initial implementation of reporting systems in Australia in 1993 [35]. Introducing students from health-related disciplines to the reporting process, emphasizing the understanding of why, how, and for what purpose they should contribute, aims to foster a safety culture among the forthcoming generations of health care professionals. Encouraging students to view errors as valuable learning opportunities rather than indicators of incompetence is highly necessary. Embracing mistakes as integral components of the learning process can foster a growth mindset where challenges become stepping stones to improvement. This positive approach not only cultivates resilience but also promotes a more constructive and proactive attitude toward learning.

Since digital systems offer a more enduring record-keeping mechanism and facilitate a higher volume of reports than their paper counterparts [9], approaches such as this one can increase the correctness and impact on the future rate of reporting. In addition, this educational practice should help overcome the initial reluctance that discourages reporting safety incidents. To know and have used an incident reporting tool, describing a safety incident and reflecting on its potential causes and the measures that could actively and thoughtfully prevent it, should have an impact on attitudes toward reporting [36,37].

### Future Research

Several scales have been developed to assess students’ knowledge and the information they receive, aiming to model their safety culture. Among these, the scales proposed by Flin et al [29] and Mira et al [38] are remarkable. However, we need to identify which mechanisms are most effective in integrating curriculum content that matches the students’ knowledge levels and attitudes, fostering a cross-disciplinary education in patient safety.

In addition, although the students scored 4.0 out of 5 regarding the fact that after this experience, they would be capable of making reports properly, a follow-up over time is required to really verify the benefits brought by this experience. Furthermore, it would be interesting to consider the use of this tool in students of earlier courses, provided they undergo some period of their training in clinical settings, to analyze its utility in earlier stages of education. This would also facilitate the development of longitudinal studies to monitor the impact in terms of reporting.

### Limitations

The aim of this experience was not to detect the safety incidents themselves but rather to train students to make correct reports in their future professional practice. Thus, the frequencies and features described in this paper did not necessarily represent the actual safety incidents occurring and what students could witness in their countries.

Recognizing an error is not straightforward. Students in training may consider it risky for their future to report an incident, leading to a restriction in the information they provide to the system. If they end up working in an environment where psychological safety is at risk, despite actively participating in this educational practice, they might choose silence, and fear of potential negative consequences could undo what was gained from this practice. The same can happen with other organizational factors that may hinder and make reporting difficult for the group of professionals in a center. This practice does not prevent this from happening in some contexts.

It cannot be guaranteed that the reports accurately reflect incidents that actually occurred. A convenience sample was used, which restricts the generalizability of the results. The medical and nursing curricula in the different participating countries were not identical. Although participation in the study was offered in the context of subjects related to patient safety, it was not possible to control for students’ baseline knowledge of incident reporting. These differences may have influenced the quality of the reports. It will be necessary to delve into the safety culture in the course of subjects with patient safety content in training programs. The constant technological evolution requires timely updating of the proposal, adapting it to possible technological solutions. Student involvement should be facilitated by the participation of academics in the project. However, the project schedule may be affected by the academic obligations of this group (eg, exams, vacations, and internship periods).

With this exercise, students become familiar with a fundamental tool in patient safety that they will encounter at the beginning of their professional careers and often approach with some hesitation, particularly in the countries where the study was conducted. However, the reports are based on observations made during their placements, and the analysis of the proposed improvement plan was conducted without accessing all the clinical information necessary for a precise analysis of root and immediate causes. In this case, the remote causes could not be determined during the exercise.

The sample size and the study’s cross-sectional nature did not allow for assessing the impact of evaluators’ feedback on students’ learning and the quality of their subsequent reports. In the future, longitudinal studies with repeated measures over time would make it possible to establish the effect of feedback.

Finally, we would have liked to establish a user-centered platform from the outset. However, due to the lack of previous information from students regarding the subject matter, it was not feasible to conduct a consultation to determine which elements to consider. We have endeavored to compensate for this by incorporating the feedback provided subsequently.

### Conclusions

In Europe, only a handful of medical or nursing schools have incorporated curriculum plans addressing patient safety [14]. Studies examining the nature of patient safety training received by students in health care disciplines are limited [39,40]. Faculties and schools might consider these reflections and data, incorporating reporting as a practical exercise into their curriculum. This study presents an initial adaptation of reporting systems to suit the needs of students, introducing a guided and inspiring framework that has garnered positive acceptance and evaluation among students. Through this endeavor, a pathway toward a safety culture within the faculty is established.

## Appendix

Multimedia Appendix 1 STROBE checklist for cross-sectional studies.

Multimedia Appendix 2 Acronym and visual identity developed for the platform.

Multimedia Appendix 3 Educational materials used during the seminars.

Multimedia Appendix 4 Schematic diagram of the Safety Incident Report System for Students platform operation.

Multimedia Appendix 5 Safety Incident Report System for Students reporting form.

## Abbreviations

AE: adverse event
BACRA: Based on Root Cause Analysis
IRS: incident reporting system
SAFEST: Safety Incident Report System for Students
STROBE: Strengthening the Reporting of Observational Studies in Epidemiology

## Data Availability

The data sets generated during and analyzed during this study are available from the corresponding author on reasonable request.
