# Supplementary figures and images for: Development and Implementation of a Safety Incident Report System for Health Care Discipline Students During Clinical Internships: Observational Study
Source: JMIR Med Educ. 2024 Jul 18;10:e56879. doi: 10.2196/56879 (PMC11294782; doi:10.2196/56879)

safest

safest

is

is

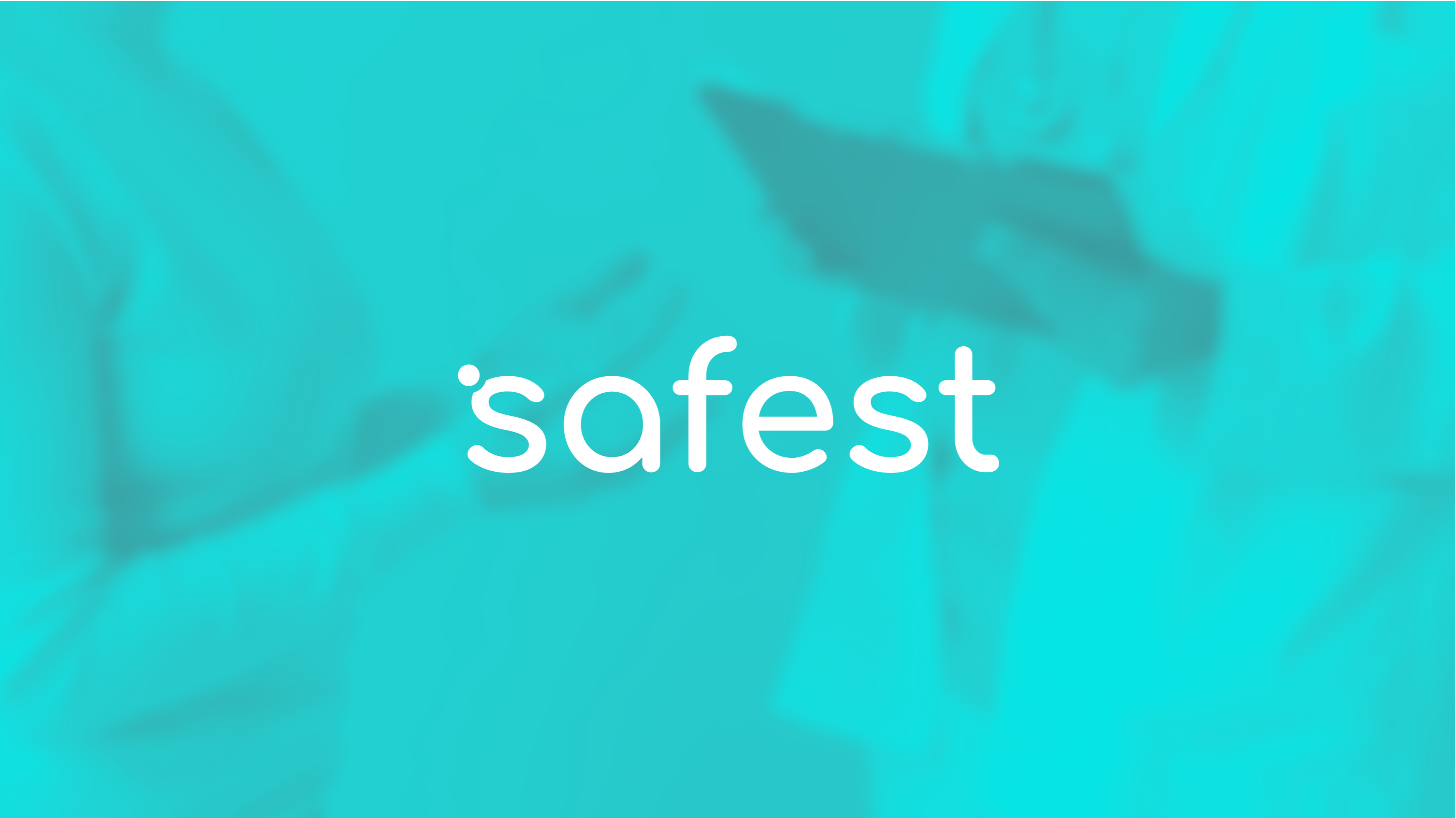

safest

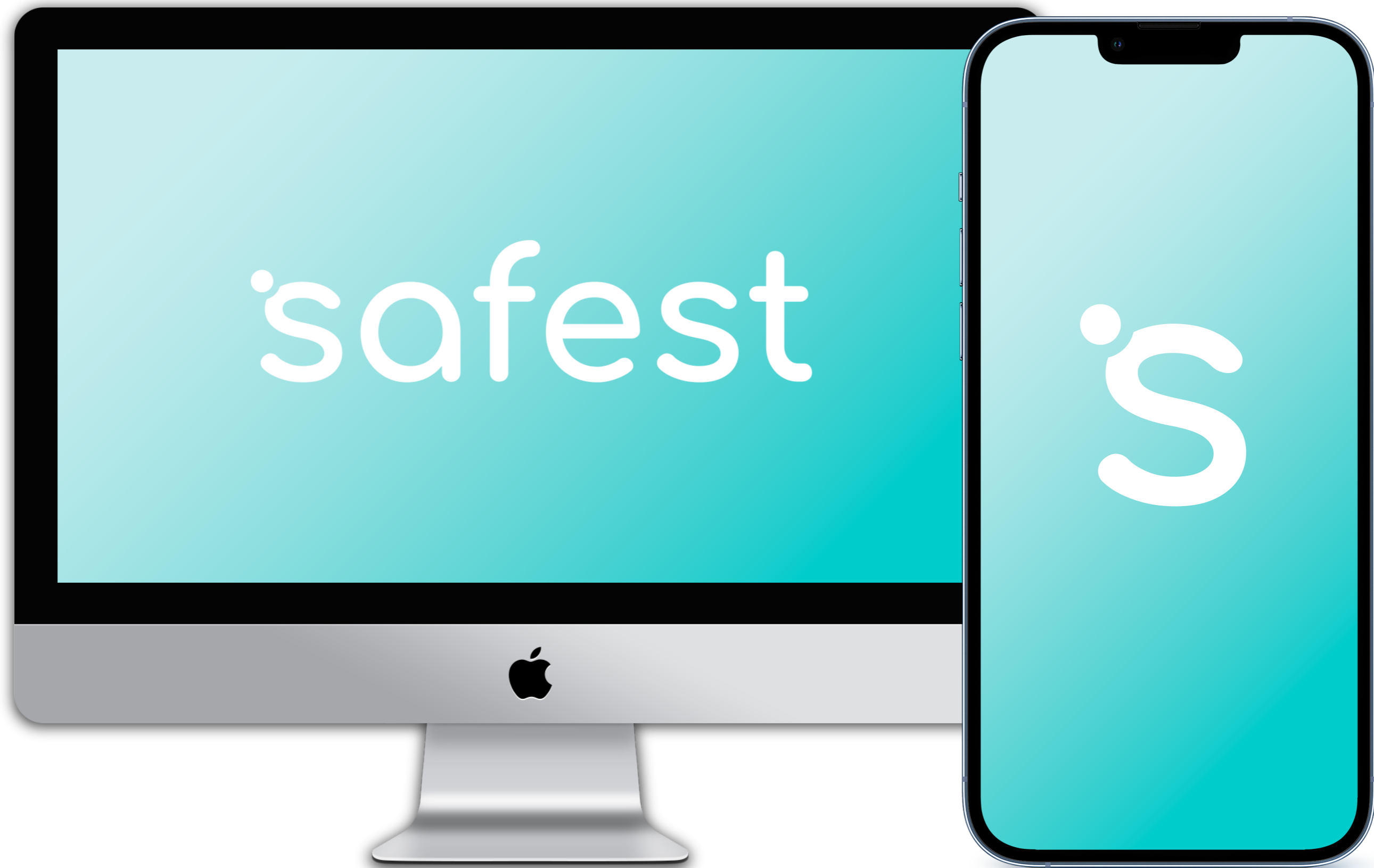

Supplement: Multimedia Appendix 2 [file mededu_v10i1e56879_app2.pdf]

## REPORTING STAGE

## GAMIFICATION STAGE

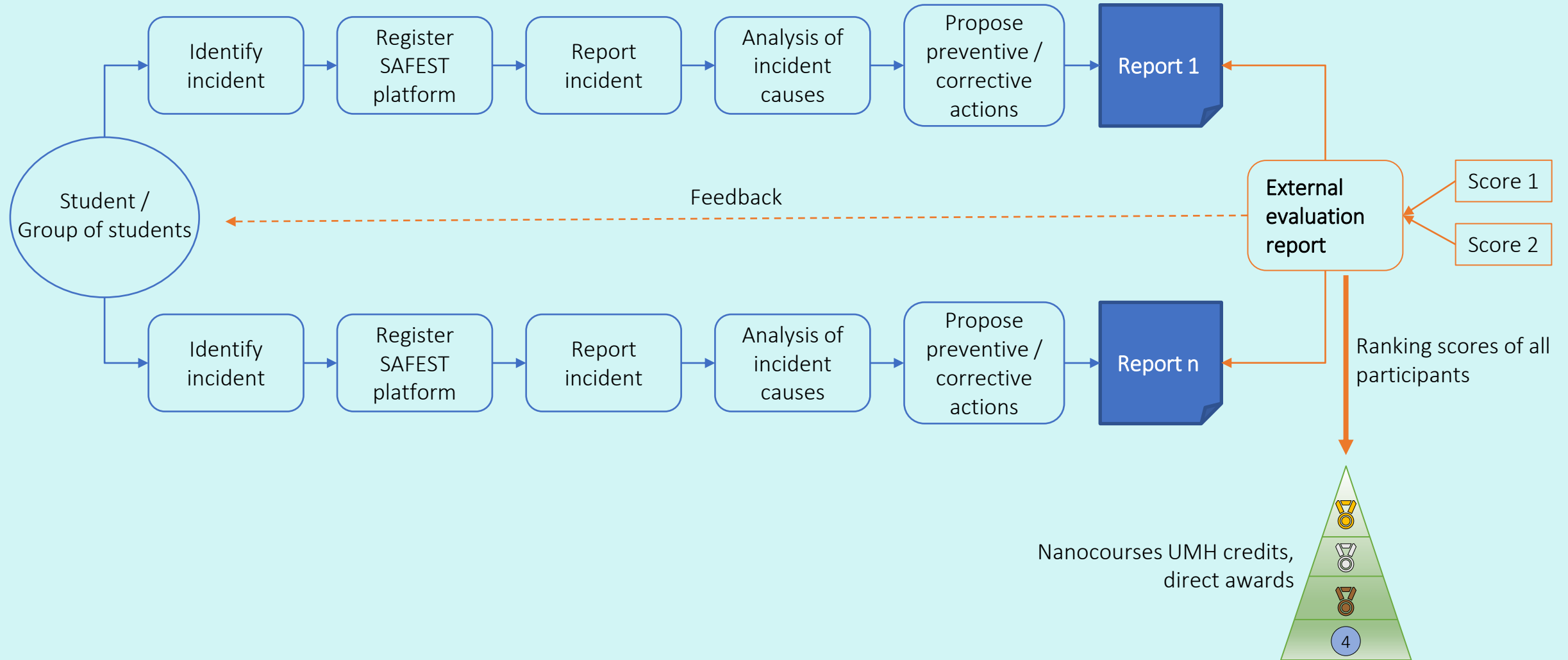

Supplement: Multimedia Appendix 4 [file mededu_v10i1e56879_app4.pdf]
