# Supplementary material for: Development and Implementation of a Safety Incident Report System for Health Care Discipline Students During Clinical Internships: Observational Study
Source: JMIR Med Educ. 2024 Jul 18;10:e56879. doi: 10.2196/56879 (PMC11294782; doi:10.2196/56879)
Supplement: Multimedia Appendix 3 [file mededu_v10i1e56879_app3.pdf]

safest

Introduction

- Everyone can make mistakes.
- Errors occur in any human activity.
- The quality systems were designed precisely to avoid/reduce errors.
- In "High Reliability Organizations" (HRO) errors have fatal consequences.
- HROs lead security actions.

Introduction

The reasonable thing is to leave better than to enter

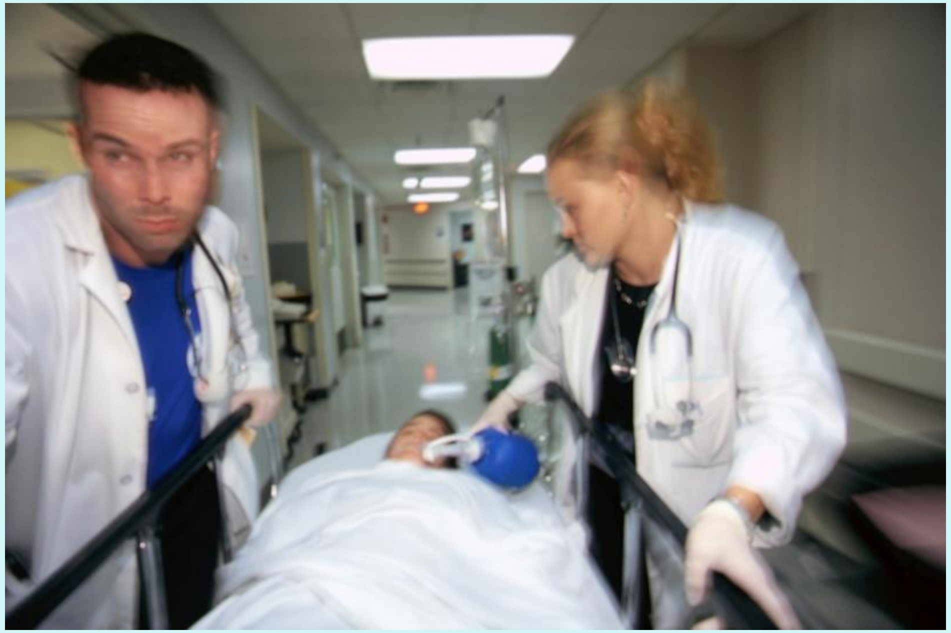

Introduction

1999

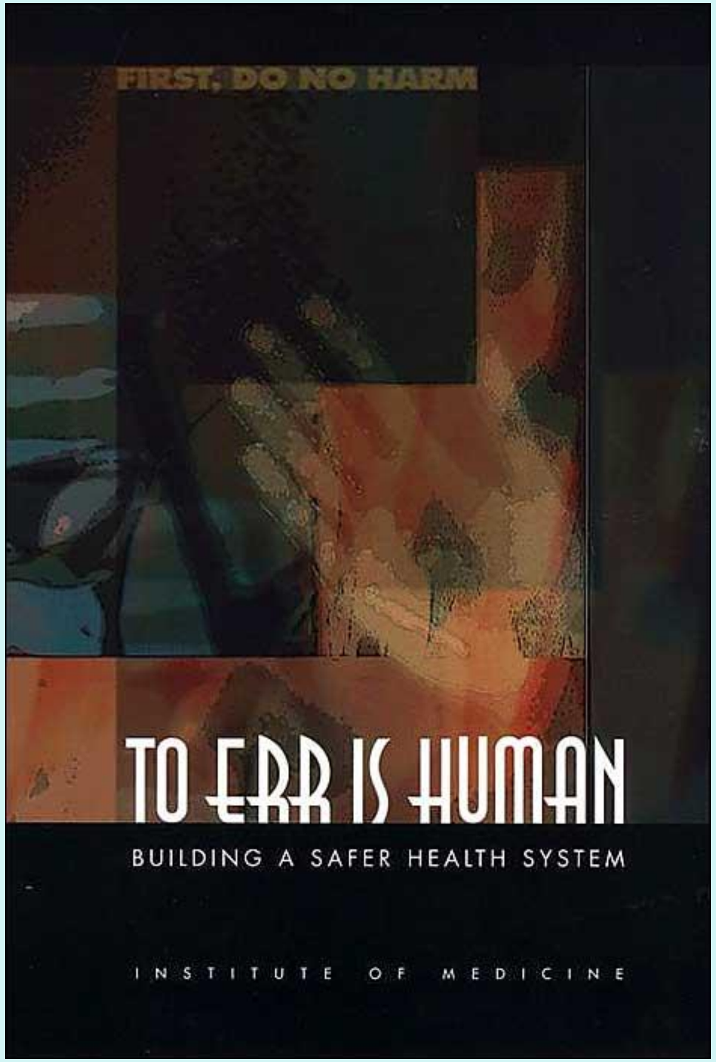

Talking about clinical errors is not easy because of their emotional, economic, social, professional and legal consequences.

But not talking about it is irresponsible.

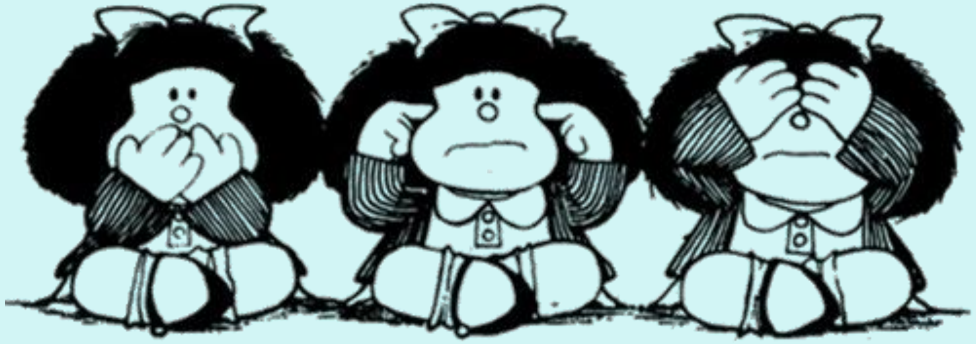

safest

What's going on?

Frequency, consequences and avoidability

Meta-analysis, 8 reference studies in USA, UK, Australia, Canada (De Vries *et al.*)

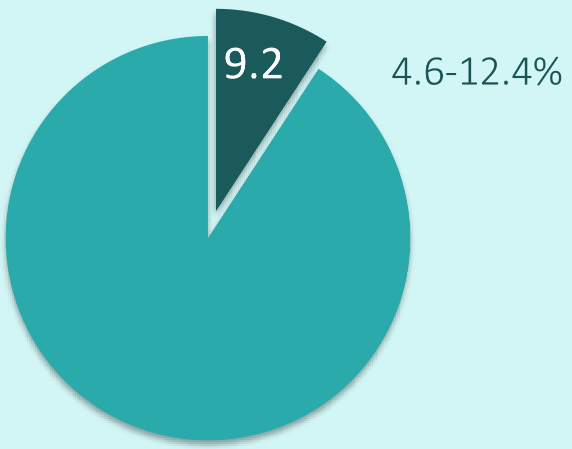

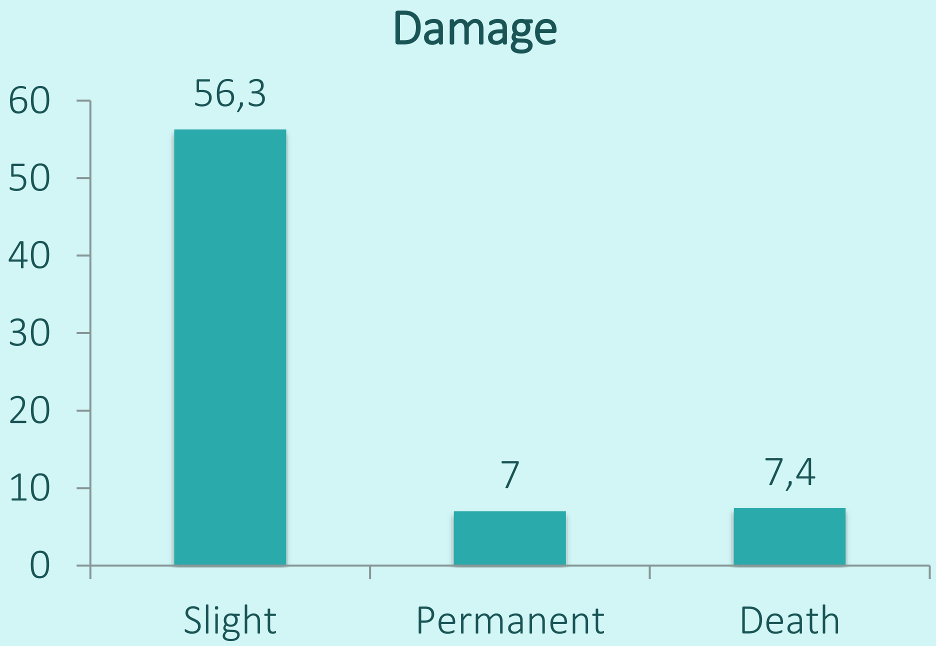

Meta-analysis, 8 reference studies in USA, UK, Australia, Canada (De Vries *et al.*)

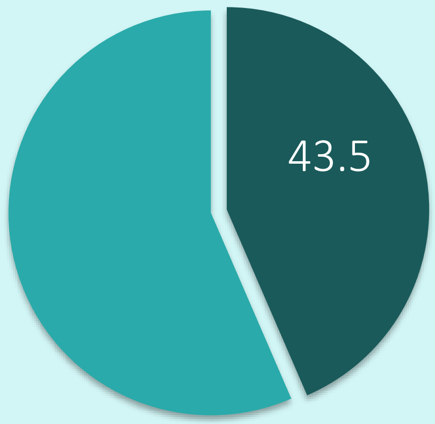

safest

Why does it happen?

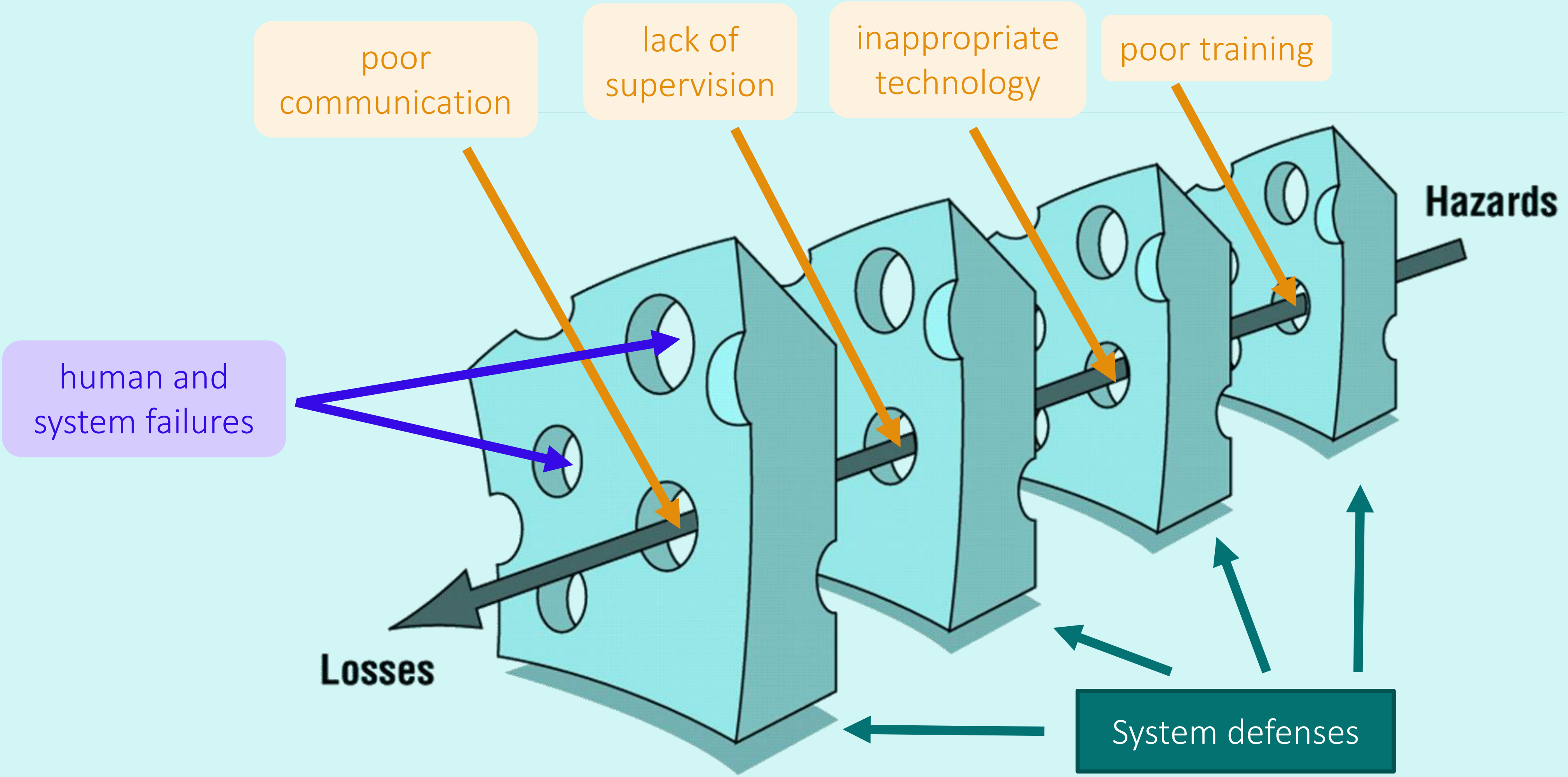

safest

Reason, 1990

How do we avoid it?

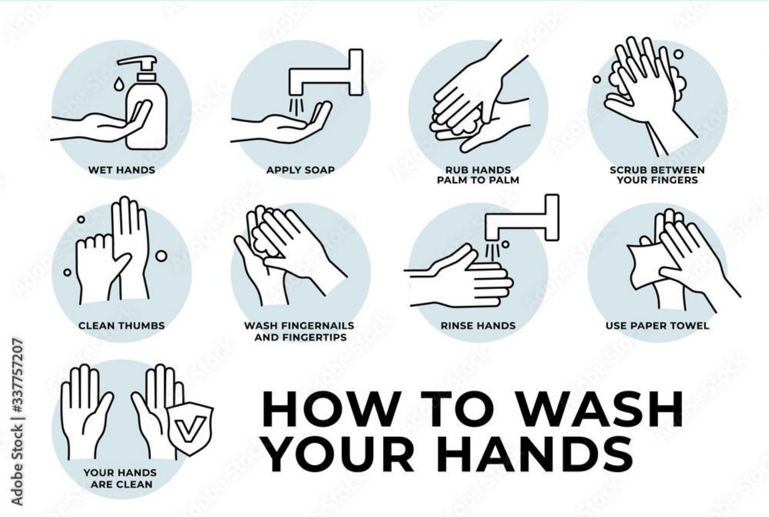

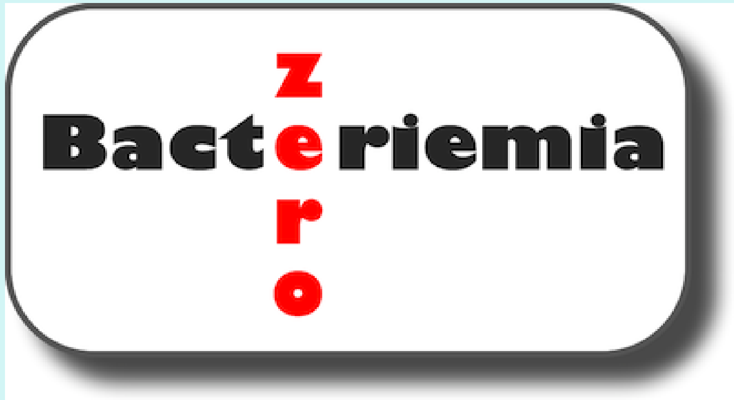

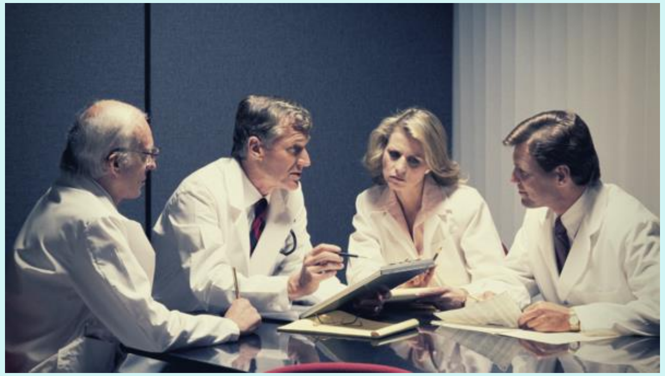

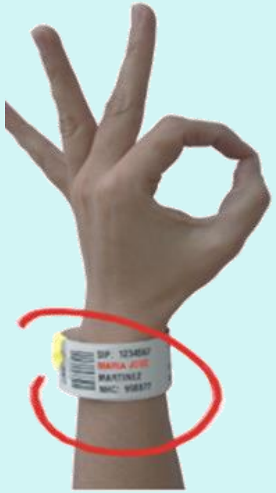

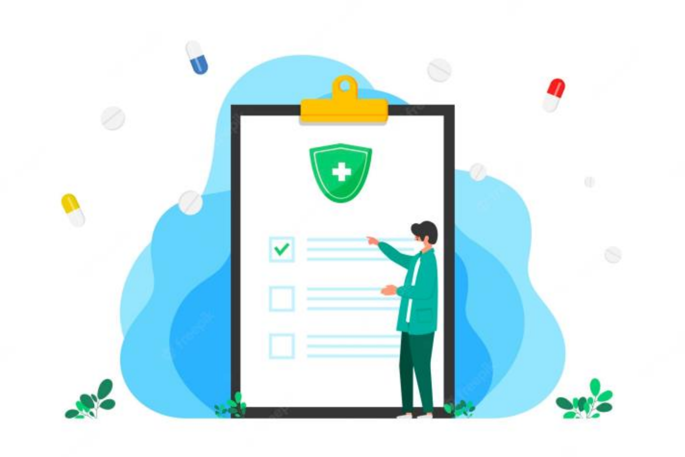

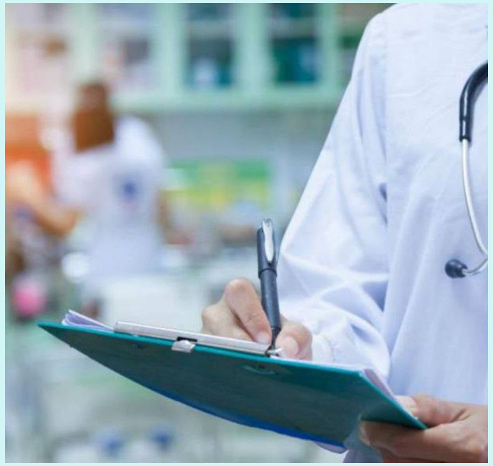

safest

How do we avoid it?

Reporting Systems

Share information → Prevent problems

Experiences, data and information

Knowledge

Learning

Sirio CA, Keyser DJ, Norman H, Weber RJ, Muto CA. Sahared learning and drive to improve patient safety: lessons learned from the Pittsburg regional healthcare iniciative. Advances in Patient Safety. Vol: 3. 2005

Incident Reporting Systems

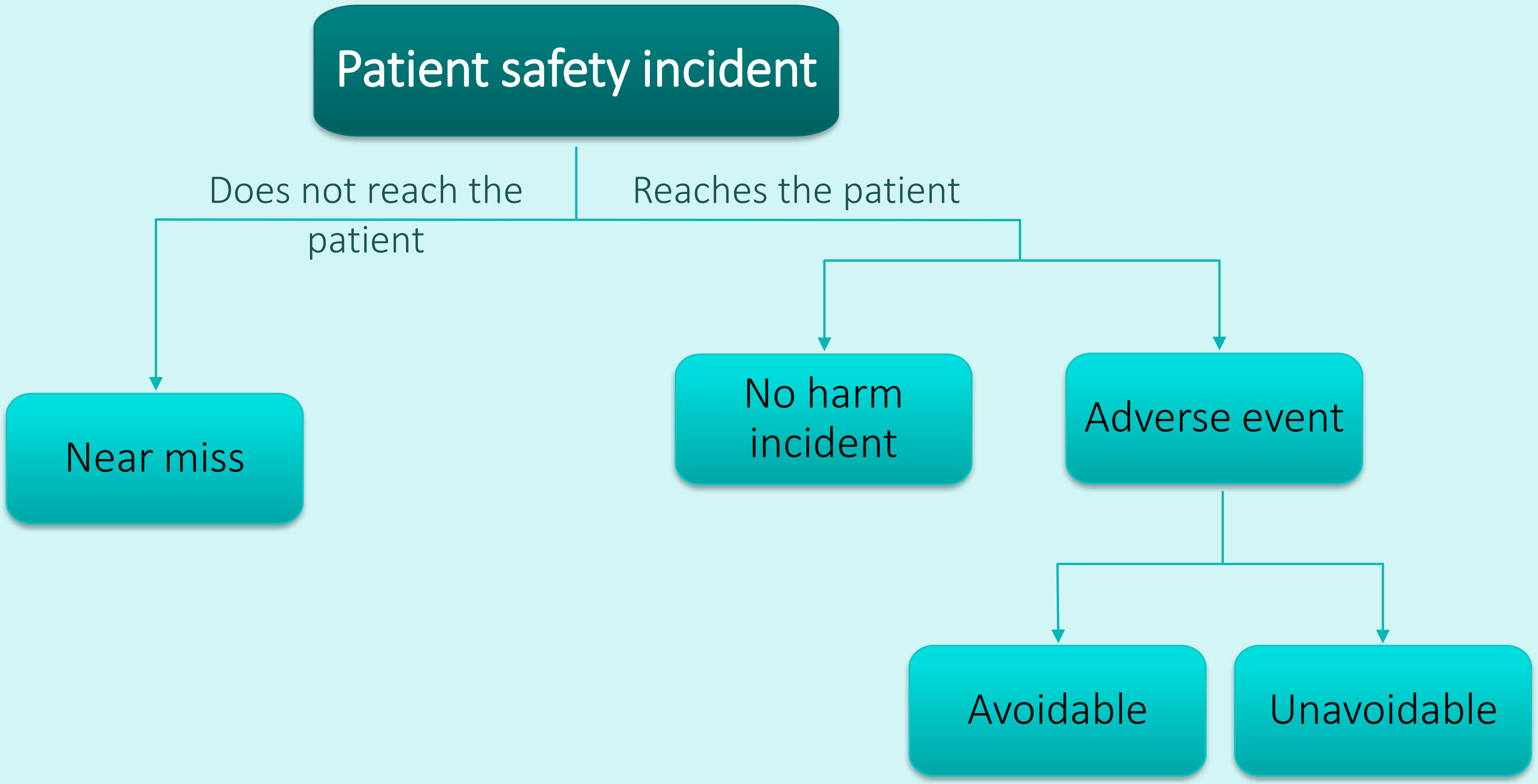

Patient safety incident reporting and learning systems: technical report and guidance. Geneva: World Health Organization; 2020

SAFEST

News

- ☑ Aimed at students of health disciplines.
- ☑ It allows participation by teams of 2-3 people.
- ☑ It includes analysis of possible causes and a proposal for preventive or corrective measures.
- ☑ Feedback on reports made.
- ☑ Benefits in the form of academic credits (UMH Nanocourse) and access to direct awards.

safest

SAFEST

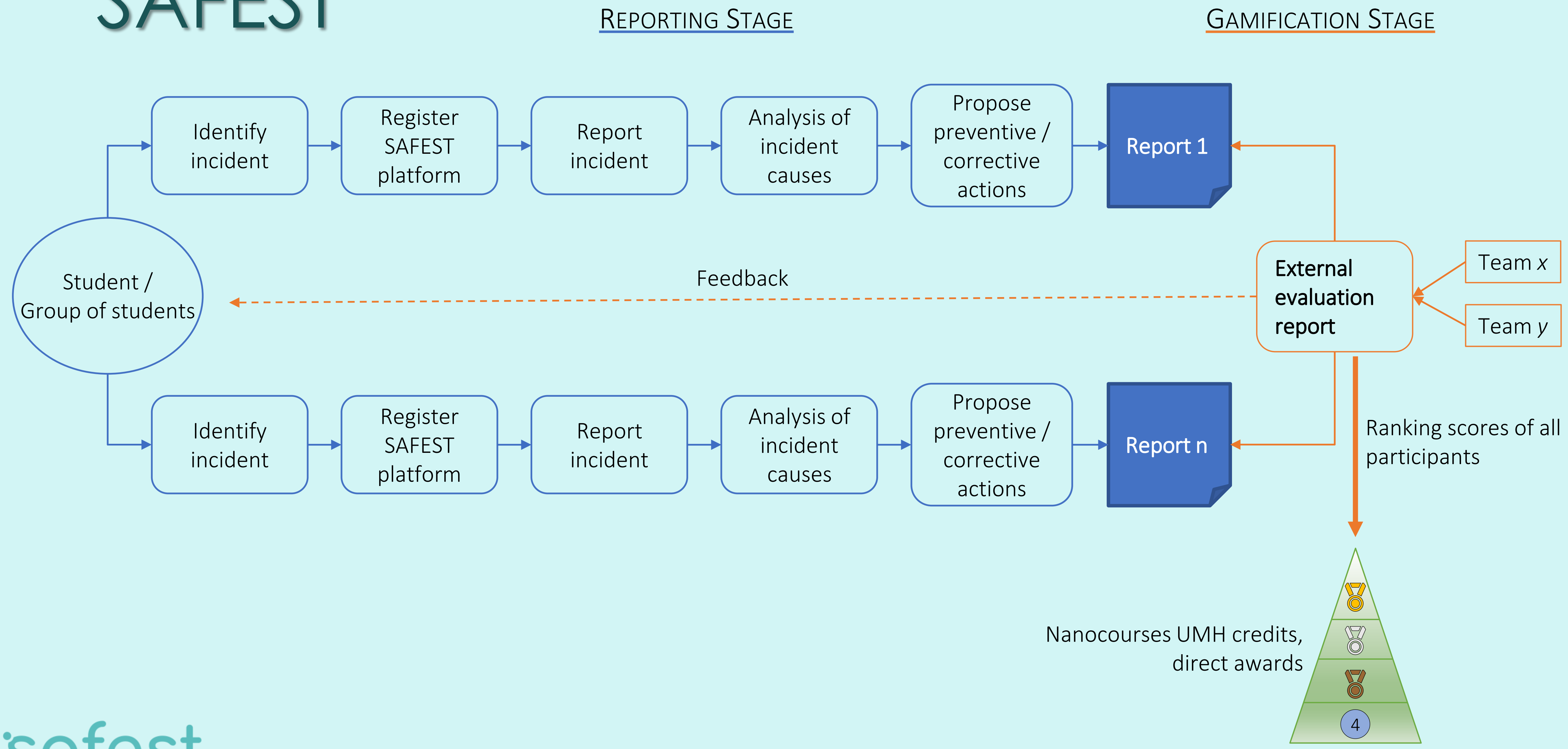

Reporting Stage
Gamification Stage
Student / Group of students
Identify incident
Register SAFEST platform
Report incident
Analysis of incident causes
Propose preventive / corrective actions
Report 1
Identify incident
Register SAFEST platform
Report incident
Analysis of incident causes
Propose preventive / corrective actions
Report n
Feedback
External evaluation report
Team x
Team y
Ranking scores of all participants
Nanocourses UMH credits, direct awards
4

safest
