# Supplementary material for: Development and Implementation of a Safety Incident Report System for Health Care Discipline Students During Clinical Internships: Observational Study
Source: JMIR Med Educ. 2024 Jul 18;10:e56879. doi: 10.2196/56879 (PMC11294782; doi:10.2196/56879)
Supplement: Multimedia Appendix 5 [file mededu_v10i1e56879_app5.docx]

**SAFETY INCIDENTS REPORT BY STUDENTS DURING THEIR CLINICAL INTERNSHIP**

Welcome to the **Safety Incident Reporting System for Students during their Clinical Internship (SAFEST)**.

Among the recommendations to increase patient safety, Incident Report Systems have a long history to identify potential sources of risk, analyzing their causes (both direct and remote) and proposing actions for better management of the risks inherent to health care. In the WHO’s *Patient safety incident reporting and learning systems: technical report and guidance* (ISBN: 9789240010338), the importance of reporting all incidents that affect patient safety is emphasized in order to reduce the risks inherent in healthcare activities and increase the quality of care.

In this experience, a collaborative virtual environment has been created to learn how to report, analyze incidents and propose improvements. It is possible to participate both individually and in groups of 2-3 students. In the latter case, the members of the group should be indicated in the comments section at the end of the questionnaire. The reports will be externally assessed considering the content of the report, the analysis of the remote and immediate causes and the proposed corrective or preventive plan. Depending on the number of reports sent, the scores obtained and the evolution, it will be possible to obtain direct rewards and recognition in nanocourses of the UMH.

To participate, the first step is to be registered by completing the form fields with the user data and a password of at least 6 characters. Once logged in, the *Dashboard* will appear, from where you can make a new report, retake a report already started, assess the reports of your colleagues and view your personal statistics.

The data provided will be used only for educational purposes.

It is important to note that if you do not complete those questions marked as Required you will not be able to continue to the next page. In addition, it is essential to click **Finish**, in the lower right corner, to submit the form definitively.

In the following links you can find an example of how to perform a notification:

- Clip 1: Introduction & register (<https://www.youtube.com/watch?v=Cp9TAsd9bsY>)
- Clip 2: Notification (<https://www.youtube.com/watch?v=0Ecnr5D9DFs>)

The study is part of the intramural project UGP-21-215 of FISABIO and is authorized by the REC Sant Joan d'Alacant University Hospital (22/027) and registered in ClinicalTrials (NCT05350345).

Thank you very much for your participation.

**DATA ON THE REPORTING CENTER**

1. Center type

- Hospital
- Primary Care Center
- Specialized Care Center

**PATIENT’S DATA**

1. Patient’s age

- Less than 28 days
- From 1 month to 1 year
- From 2 to 4 years
- From 5 to 11 years
- From 12 to 17 years
- From 18 to 25 years
- From 26 to 35 years
- From 36 to 45 years
- From 46 to 55 years
- From 56 to 65 years
- From 66 to 75 years
- From 76 to 85 years
- More than 85 years

1. Patient’s sex
   - Man
   - Women
   - Other
2. Patient’s risk factors
   - Alcoholism
   - High cholesterol
   - Comorbidity
   - Culture/belief that makes proper handling difficult
   - Cognitive deficit or mental disorder
   - Dependence
   - Age
   - Diseases that affect the immune system
   - Fragility
   - Hyperglycemia
   - Impaired mobility
   - Polypharmacy
   - High blood pressure
   - Sedentary lifestyle
   - Overweight or obesity
   - Smoking
   - Other: _________________________

**NOTIFIERS’S DATA**

1. Notifier’s sex
   - Man
   - Woman
   - Other
2. Country from which the notification is made
   - Spain
   - Germany
   - Chile
   - Colombia
   - Croatia
   - Ecuador
   - Slovakia
   - Estonia
   - Finland
   - Israel
   - Latvia
   - Lithuania
   - Portugal
   - Other: _________________________
3. Studies in course

- University
  - Degree
  - Bacteriology
  - Biology
  - Biochemistry
  - Biotechnology
  - Food Science and Technology
  - Biomedical Sciences
  - Nursing
  - Pharmacy
  - Physiotherapy
  - Genetics
  - Surgical Instrumentation
  - Speech Therapy
  - Medicine
  - Microbiology
  - Nutrition and Dietetics
  - Odontology
  - Optics and Optometry
  - Psychology
  - Occupational Therapy
  - Other: _________________________
  - Master’s degree
  - Osteopathic Approach to the Locomotor System
  - Sanitary Humanitarian Action
  - Therapeutic Physical Activity
  - Therapeutic Physical Activity for People with Chronic Pathology, Aging or Disability
  - Sanitary Administration
  - Food and Health
  - Food, Nutrition and Metabolism
  - Biological Analysis and Laboratory Diagnosis
  - Analysis of Omic Data
  - Functional Analysis in Clinical and Health Contexts
  - Sanitary Analysis
  - Emergency and Disaster Analysis and Management
  - Anatomy Applied to the Clinic
  - Medical Anthropology and International Health
  - Health Assistance and Research
  - Clinical and Basic Aspects of Pain
  - Emotional Care for Hospitalized Children
  - Pharmaceutical Care
  - Pharmaceutical Care in Geriatrics
  - Pharmaceutical Care and Healthcare Pharmacy
  - Pharmaceutical Care and Pharmacotherapy
  - Physiotherapic Care in Physical Activity and Sport
  - Comprehensive Care for the Critically Ill and Emergencies
  - Palliative Care for People with Advanced Illnesses
  - Healthcare and Collaborative Practice
  - Healthcare, Management and Care
  - Early Attention
  - Palliative Care
  - Audiology
  - Advances in Cardiology
  - Advances in Research and Treatments in Psychopathology and Health
  - Advances in Neurorehabilitation of Communicative and Motor Functions
  - Advances in Diagnostic and Therapeutic Radiology and Physical Medicine
  - Advances in Radiology and Physical Medicine
  - Bases for the Care and Education of People with Diabetes
  - Biobanks and Use of Human Biological Samples in Biomedical Research
  - Bioethics
  - Bioethics and Biolaw
  - Sanitary Biology
  - Biomechanics Applied to Damage Assessment
  - Biomechanics and Sports Physiotherapy
  - Health Biotechnology
  - Biotechnology in Health Sciences
  - Medical Biotechnology
  - Biotechnology for Health and Sustainability
  - Sanitary Biotechnology
  - Pharmaceutical Science and Technology and Rational Use of Medication
  - Sciences Applied to the Prevention and Functional Rehabilitation of Sports Injuries
  - Nursing Sciences
  - Health Sciences: Research and New Challenges
  - Nervous System Sciences:Neurotoxicology, Neuropsychopharmacology, Neuromusculoskeletal Physiotherapy, Neurorehabilitation
  - Pharmaceutical Sciences
  - Medical and Health Sciences
  - Dental Sciences
  - Dental and Stomatological Sciences
  - Social Sciences Applied to Social and Health Care
  - Oral Surgery and Implantology
  - Advanced Urological Minimally Invasive Surgery
  - Advanced Oral Surgery and Implantology
  - Oral Surgery and Implantology
  - Oncologic Surgery
  - Podiatric Surgery
  - Minimal Incision Podiatric Surgery for Podiatrists
  - Advanced Medical Competencies
  - Nutrition and Health Communication
  - Cornea and Contact Lenses
  - Chronicity and Dependence
  - Critical Care
  - Long Term Nursing Care
  - Nursing Care in Dialysis and Renal Transplantation
  - Intensive Nursing Care
  - Health Care, Disability, Dependency and End of Life
  - Health Care for the Promotion of the Autonomy of People and Attention to End Processes
  - Nursing Care. Methodology and Applications
  - Comprehensive Nursing Care in Nephrological Processes
  - Comprehensive Nursing Care in Critical Situations and Urgencies in Adults
  - Palliative Care
  - Pediatric Palliative Care
  - Culture of Care
  - Deterioration of Skin Integrity, Ulcers and Wounds
  - Imaging Diagnostic in Cardiology
  - Disability and Dependency
  - Craniofacial Pain and Dysfunctioncervicocraniomandibular
  - Orofacial Pain and Craniomandibular Dysfunction
  - Diagnostic and Therapeutic Cardiac Electrophysiology
  - Emergencies and Catastrophes
  - Endodontics
  - Advanced Endodontics
  - Endodontics and Restorative Dentistry
  - In-Hospital Cardiology Nursing
  - Anesthesia Nursing
  - Critical Care Nursing, Urgencies and Emergencies
  - Advanced Practice Nursing in Chronicity and Dependency Care
  - Advanced Practice Nursing for the Care of Complex Chronic Patients
  - Advanced Clinical Practice Nursing
  - Occupational Health Nursing
  - Urgency and Critical Care Nursing
  - Urgency and Emergency Nursing
  - Urgencies, Emergencies and Special Care Nursing
  - Nursing in Critical Care and Urgencies
  - Nursing in Palliative Care
  - In-Hospital Urgencies and Critical Care Nursing
  - Nursing in Urgencies, Emergencies and Health Transport
  - School Nursing and Health Consulting in Educational Centers
  - Ophthalmological Nursing
  - Ophthalmic Nursing and Vision Sciences
  - Oncology Nursing
  - In-Hospital Pediatric and Neonatal Nursing
  - Optometric Clinical Specialties
  - Specialization in Nursing Care
  - Specialization in Endodontics
  - Specialization in Logopedic Intervention
  - Professional Specialization in Pharmacy
  - Dental Aesthetics
  - Evaluation and Physical Training for Health
  - Neuropsychological Evaluation and Rehabilitation
  - Healthcare Pharmacy and Pharmaceutical Care
  - Medical Physics
  - Physiology
  - Integrative Physiology
  - Physiology and Neuroscience
  - Physiotherapy Applied to Sport and Physical Activity
  - Physical Activity and Sports Physiotherapy
  - Physiotherapy of the Aging Processes: Social and Health Strategies
  - Sports Physiotherapy and Readaptation to Physical Activity
  - Physiotherapy of the Musculoskeletal System
  - Chest Physiotherapy
  - Sports Physiotherapy
  - Physiotherapy in the Comprehensive Approach to the Pelvic Floor
  - Physiotherapy in the Neurological Approach of Children and Adults
  - Physiotherapy in Sport
  - Physiotherapy in Physical Activity and Sport
  - Physiotherapy in Pediatrics
  - Invasive Physiotherapy
  - Manual Physiotherapy of the Musculoskeletal System
  - Advanced Musculoskeletal Physiotherapy Based on Clinical Reasoning
  - Neurological Physiotherapy
  - Neurological Physiotherapy of Children and Adults
  - Neurological Physiotherapy: Assessment and Treatment Techniques
  - Neuromusculoskeletal Physiotherapy
  - Pediatric Physiotherapy
  - Respiratory and Cardiac Physiotherapy
  - Physiotherapy and Disability
  - Physiotherapy and Scientific Evidence
  - Physiotherapy and Rehabilitation in Sport
  - Geriatrics and Gerontology: Comprehensive Dependency Care
  - Gerontology
  - Nursing Care Management
  - Quality Management in Health Services
  - Management of Clinical Patient Safety and Quality of Health Care
  - Health Management
  - Management and Application of Knowledge of Self-care in Nursing
  - Management and Care in the Pharmacy Office
  - Diagnostic Cardiac Imaging
  - Advanced Oral Implantology
  - Oral Implantology and ProstheticsImplant supported
  - Intervention for People with Alzheimer’s Disease
  - Intervention and Research in Language Pathology
  - Intervention in Disability and Dependence
  - Speech Therapy Intervention
  - Specialized Speech Therapy Intervention
  - Clinical Analisys Laboratory
  - Leadership and Management of Nursing Services
  - Medicines, Health and Health System
  - Urgency and Emergency Medicine
  - Evaluative Medicine and Disability in the Locomotor System
  - Respiratory Medicine
  - Translational Medicine
  - Tropical Medicine and Development Cooperation
  - Menopause
  - Microbiology
  - Applied Microbiology
  - Microbiology Applied to Public Health and Research in Infectious Diseases
  - Advanced Microbiology
  - Microbiology and Parasitology: Research and Development
  - Microbiology and Health
  - Reconstructive Microsurgery
  - Motor Neurocontrol
  - New Care Trends in Health Sciences
  - Nutrition
  - Nutrition Applied to Health
  - Clinical Nutrition
  - Clinical and Community Nutrition
  - Nutrition in Physical Activity and Sport
  - Human Nutrition
  - Human Nutrition and Food Quality
  - Human Nutrition and Applied Dietetics
  - Personalized and Community Nutrition
  - Nutrition and Food
  - Nutrition and Human Food
  - Nutrition and Metabolism
  - Nutrition and Health
  - Nutrigenomics and Personalized Nutrition
  - Children’s Dentistry, Orthodontics and Community Dentistry
  - Integrated Dentistry
  - Medical-Surgical and Comprehensive Dentistry
  - Aesthetic Restorative Dentistry
  - Restorative, Aesthetic and Functional Dentistry
  - Pediatric Dentistry
  - Molecular Oncology
  - Ocular Oncology, Orbit and Oculoplastics
  - Optics and Advanced Optometry
  - Optometry
  - Advanced Optometry
  - Advanced Optometry and Vision Sciences
  - Advanced Optometry and Visual Health
  - Clinical Optometry
  - Advanced Clinical Optometry and Research
  - Clinical Optometry and Advanced Optics
  - Clinical Optometry and Vision
  - Optometry and Clinical Research
  - Optometry and Vision Sciences
  - Optometry and Vision
  - Orthodontics
  - Advanced Orthodontics
  - Comprehensive Orthodontics
  - Orthodontics and Orthopedics Dentofacial
  - Osteopathy
  - Structural Osteopathy
  - Integrative Osteopathy
  - Pediatric Osteopathy
  - Osteopathy and Manual Therapy
  - Advanced Periodontics
  - Surgical Medical Periodontics
  - Clinical Podiatry and Advanced Podiatric Surgery
  - Sports Podiatry
  - Advanced Practice and Management in Nursing
  - Prevention and Rehabilitation of Sports Injuries
  - Prosthesis, Implant Prosthesis and Dental Aesthetics
  - Medical Chemistry
  - Functional Recovery in Physiotherapy
  - Rehabilitation of the Neurological Patient
  - Neuropsychological Rehabilitation and Cognitive Stimulation
  - Psychosocial Rehabilitation in Community Mental Health
  - Visual Rehabilitation
  - Retina
  - Cardiovascular Risk
  - Public Health
  - Public Health in Disasters
  - Public Health and Health Management
  - Regional Sustainable Health Systems
  - Ophthalmological Subspecialties
  - Advanced Physiotherapy Techniques for Cancer Patients
  - Advanced Techniques of Respiratory and Cardiac Physiotherapy
  - Advanced Techniques in Physiotherapy for Women
  - Advanced Aesthetic and Laser Techniques
  - Endoscopic Surgical Techniques
  - Optical and Imaging Technologies
  - Telemedicine
  - Orthopedic Manual Therapy in the Treatment of Pain
  - Orthopedic Manual Therapy: General and Specific Methods
  - Osteopathic Manual Therapy
  - Evidence-Based Occupational Therapy: Study of Upper Limb Functionality
  - Occupational Therapy in Neurology
  - Manual Therapies
  - Non-Pharmacological Therapies in Dementias
  - Sanitary Social Work
  - Communication Disorders
  - Communication and Language Disorders
  - Voice and Language Disorders
  - Eating Disorders and Obesity
  - Support Treatment and Palliative Care in the Oncological Patient
  - Sports Traumatology
  - Adult Urgencies and Emergencies for Nursing Professionals
  - Urgencies, Emergencies and Catastrophes
  - Urgencies, Emergencies and Critical in Nursing
  - Assessment of Bodily Injury, Brain Injury and Disabilities
  - Assessment and Care for People with Physical Disabilities
  - Other: _________________________
- Non-university
  - Medium Grade Training Cycle
  - Care for People in a Dependency Situation
  - Nursing Auxiliary Care
  - Sanitary Emergencies
  - Pharmacy and Parapharmacy
  - Administrative Management
  - Electrical and Automatic Installations
  - Laboratory Operations
  - Other: _________________________
  - Superior Grade Training Cycle
  - Physical Conditioning
  - Administration and Finance
  - Pathological Anatomy and Cytodiagnosis
  - Prosthetic Audiology
  - Dietetics
  - Sanitary Documentation and Administration
  - Clinical Electromedicine
  - Socio-sports Teaching and Animation
  - Manufacture of Pharmaceutical, Biotechnological and Related Products
  - Oral Hygiene
  - Imaging for Diagnostic and Nuclear Medicine
  - Social Integration
  - Clinical and Biomedical Laboratory
  - Laboratory of Analysis and Quality Control
  - Electronic Maintenance
  - Orthotics and Support Products
  - Professional Risk Prevention
  - Promotion of Gender Equality
  - Dentures
  - Radiotherapy and Dosimetry
  - Environmental Health
  - Other: _________________________

1. Institution
   - Pontificia Universidad Católica de Chile
   - Universidad Austral
   - Universidad de Alicante
   - Universidad Miguel Hernández
   - Universidad de Santander
   - UNIFESP - Universidade Federal de São Paulo
   - UNIR - Universidad Internacional de La Rioja
   - Other: _________________________
2. Year

*Answer:*

1. Internship hours carried out so far in that department

*Answer*:

**INCIDENT DATA**

1. Date of the incident (dd-mm-yyyy)

*Answer:*

1. Time of the incident (hh:mm)

*Answer:*

1. Date of the notification (dd-mm-yyyy)

*Answer:*

1. Time of the notification (hh:mm)

*Answer:*

1. Where did it take place?

- Hospital
- Primary Care Center / Specialized Care Center
- In a different organization/place
- Unknown place

15.1. *If “Hospital”*: Area

- - Outpatient Department
  - Emergencies
  - Day Hospital
  - Hospitalization Units
  - Surgical Block
  - Intensive Care Units
  - Central Services
  - Delivery Room
  - Outpatient Emergency Service / Health Transport
  - Support Services
  - Other: _________________________

15.2. *If “Hospital”:* Service

*Answer:*

15.3. *If “Primary Care Center / Specialized Care Center”:* Area

- - Outpatient Department
  - Telephone
  - Administration / Information Area
  - Waiting room
  - Area of Continuous Care / Emergencies / Primary Care Emergency Service
  - Laboratory
  - Radiology
  - Other areas of the PC center (stairs, entry…)
  - Medical transport

15.4. *If “In a different organization/place”:* Place

- - Patient's Address
  - Community Pharmacy
  - On the street
  - Residence
  - Other: _________________________

1. Number of people related to the incident

*Answer:*

1. Position(s) of the person(s) involved (indicate profession(s), if applicable)
   - Health professionals: _________________________
   - Other sector workers: _________________________
   - Emergency services and security forces personnel: _________________________
   - Students / Interns: _________________________
   - Other patients - relatives - volunteers – caregivers: _________________________
   - Unknown / not applicable: _________________________
   - Others: _________________________
2. Frequency / Probability of recurrence
   - Very rare
   - Infrequent
   - Occasional
   - Probable
   - Frequent
3. Participation in the incident
   - I participated in the incident
   - I have seen the incident committed
   - I have heard about the incident

**DESCRIPTION OF THE INCIDENT**

*This section shows the possible damage caused grouped by blocks. Several options can be selected from the same block and it is not necessary to select in all blocks.*

1. Care complications
   - Falls and consequent fractures
   - Contusion
   - Acute pulmonary edema and respiratory failure
   - Phlebitis
   - Respiratory insufficiency
   - Sciatic injury
   - Other consequences of prolonged immobilization
   - Burns or erosions (diabetic foot)
   - Pressure ulcer
   - Other: _________________________
2. Care-related infection
   - Device-associated bacteremia
   - Colonization/infection with multi-resistant micro-organisms
   - Surgical site or traumatic wound infection
   - Pressure ulcer infection
   - Urinary tract infection
   - Opportunistic infection
   - Pneumonia (includes by aspiration)
   - Sepsis or septic shock
   - Other: _________________________
3. Effects of medication or medical products
   - Altered heart rhythm or electrical activity
   - Altered kidney function (nephropathy)
   - Neurological alteration
   - Headache
   - Electrolyte imbalance
   - Dehydration
   - Malnutrition
   - Dispensing of a medication that does not correspond to the patient
   - Pain (ineffective analgesia)
   - Prescription error
   - Transfusion error
   - Administration route error
   - Stupor or disorientation
   - Altered blood glucose
   - Bleeding, epistaxis, bruising
   - Hepatotoxicity
   - Hypotension
   - Inadequate glycemic control
   - Inadequate pain management
   - Acute myocardial infarction, cerebrovascular accident, pulmonary thromboembolism, deep vein thrombosis
   - Heart failure and shock
   - Drug intoxication
   - Nausea, vomiting or diarrhea
   - Neutropenia
   - Non-administration of a treatment
   - Pruritus, rash or reactive skin lesions
   - Allergic reaction
   - Contact skin reaction
   - Urinary retention
   - Ulcus or upper gastrointestinal bleeding
   - Other: _________________________
4. Complications of a procedure
   - Adhesions and functional alterations
   - Anesthetic complications
   - Local complications from radiotherapy
   - Neonatal complications from birth
   - Foreign body after an intervention
   - Vaginal tearing
   - Suture dehiscence
   - Excessive dose of radiotherapy
   - Eventration or evisceration
   - Extravasation
   - Phlebitis
   - Hematuria
   - Hemorrhage or hematoma
   - Heart failure/impairment
   - Kidney failure/impairment
   - Surgical intervention in the wrong location
   - Lacerations
   - Injury during probing
   - Organ injury
   - Pneumothorax
   - Tympanic perforation
   - Erroneous patient testing (e.g., unnecessary radiation)
   - Urinary retention
   - Delay in diagnostic test
   - Delay in vital treatment
   - Seroma
   - Surgical intervention suspension
   - Circulatory disorder (splint adjustment)
   - Pulmonary embolism
   - Other: _________________________
5. Other
   - Unexpected death
   - Worse evolutionary course of the main pathology
   - Permanent loss of function
   - Patient suicide
   - No harm
   - Other: _________________________

**DAMAGE ASSESSMENT**

1. Damage type
   - Physical
   - Psychological
   - Social
   - Unknown
   - Other: _________________________
2. Severity
   - Very serious
   - Serious
   - Moderate
   - Mild
   - Has not reached the patient
3. Patient autonomy
   - Prevents the patient from fending for himself
   - Partial limitation of self-sufficiency
   - Does not limit patient activity
4. Anticipation of the damage duration
   - Temporary
   - Permanent
   - The patient has not been affected

**FACTORS THAT HAVE CONDITIONED THE INCIDENT**

In this section the factors are grouped in blocks. Several factors can be selected from the same block and it is not necessary to select from all blocks.

1. Patient or family factors
   - Non-cooperative attitude (non-compliance)
   - Lack of family or support networks
   - Low economic level
   - Comorbidity or complexity of the condition
   - Altered cognitive status
   - Other patient’s factors
   - Educational and social factors to consider
   - Recent surgery
   - Poor communication with relatives
   - Does not provide correct or enough information
   - Mental disorder
2. Equipment and resources factors
   - Improper storage or accessibility
   - Expiration
   - Improper calibration
   - Lack of alternative materials
   - Equipment deficit (including non-sterile material)
   - Inadequate resource design (bell…)
   - Similar container or name
   - Non-standard equipment
   - New equipment or resource
   - Incorrect labeling
   - Failure or unavailability to access to the digital medical record
   - Malfunctions
   - Product/drug unavailability
   - Equipment maintenance issues
3. Individual factors of the healthcare professional(s)
   - Uncooperative attitude
   - Inadequate or insufficient anamnesis, examination, or tests
   - Low motivation
   - Confusion, oversight, distractions
   - Inadequate or insufficient knowledge or skills
   - Lack of knowledge of regulations or protocols of performance
   - Medication error (prescription or dispensing)
   - Diagnostic error
   - Inadequate or insufficient training
   - Inadequate timetable
   - Inappropriate interpretation of analytical or test results
   - Not verifying the treatment that the patient is currently taking
   - Little experience in the workplace
   - Overload, work pressure
4. Work environment factors
   - Distractions in the environment
   - Inadequate environment (cleaning, beds, space)
   - Inadequate environment (noise, light, temperature)
   - Shift-related fatigue
   - High care pressure
   - Inadequate staff/patient ratio
   - Performance of outside tasks
   - Excessive staff turnover/inexperience
   - Security and access to restricted areas
5. Oral and written communication between professionals factors
   - Ambiguous verbal indications
   - The information does not reach the entire team
   - Inappropriate body language
   - Insufficient or inadequate records
   - Using an inappropriate channel
   - Incorrect use of language
6. Patient communication factors
   - Language barrier
   - Ambiguous verbal indications
   - Inappropriate body language
   - Insufficient or inadequate records
   - Using an inappropriate channel
   - Incorrect use of language
7. Teamwork and leadership factors
   - Inaccurate assignment of tasks
   - Conflict between team members
   - Lack of coordination in the team
   - Low risk awareness
   - Inadequate supervision
   - There is no effective leadership
8. Task-related factors
   - Absence of guidelines or protocols
   - Absence of process verification
   - Inadequate or outdated protocol
   - Unknown protocol / non-compliance
   - Too complex task
9. Organizational and management factors
   - Absence of support mechanisms in a risk situation
   - Absence of evaluation systems
   - Wrong appointment or scheduling
   - Delays in the performance of tests or inter-consultations
   - Error in medical documentation
   - Error in health information
   - Insufficient care structure
   - Insufficient organizational structure
   - Inadequate or non-existent treatment plan
   - Incorrect patient identification
   - Non-existence or inadequate risk management
   - Insufficient deployment of a proactive security culture
   - Gaps or failures in the information system
   - Long waiting list
10. Other factors

*Answer:*

**CARE RECEIVED AFTER THE INCIDENT**

1. Care received after the incident
   - Health care was not affected
   - A higher level of observation and monitoring was required
   - An additional test (x-ray, culture, …) or other procedure was required
   - Medical treatment or rehabilitation (antibiotics, cures, …)
   - Additional surgery
   - Life-sustaining intervention or treatment (orotracheal intubation, CPR, ...)
   - Other: _________________________

**REFLECTIONS**

1. Has the center been notified?
   - Yes
   - No
   - I do not know
2. Could the incident have been prevented?
   - Yes
   - No *[Skip to No. 43]*
3. How could it have been prevented?

*Answer:*

1. How could the probability of occurrence or the severity of this event be reduced?

*Answer:*

1. To what extent was all the information necessary to analyze the causes of the event available?

*Answer:*

1. Have measures been put in place to prevent it from happening in the future?
   - Yes
   - No *[Skip to No. 47]*
2. What measures have been put in place to prevent this from happening in the future?

*Answer:*

1. Do you consider that the analysis could have been different if you had had access to another source of information?

*Answer:*

1. Write here any other comments you may have

*Answer:*
